# Post-translational modifications within tau paired helical filament nucleating motifs perturb microtubule interactions and oligomer formation

**DOI:** 10.1016/j.jbc.2021.101442

**Published:** 2021-11-24

**Authors:** Diana M. Acosta, Chiara Mancinelli, Clay Bracken, David Eliezer

**Affiliations:** 1 Department of Biochemistry and Program in Structural Biology, Feil Family Brain and Mind Research Institute, Weill Cornell Medicine, New York, New York, USA; 2 Department of Biochemistry and Program in Structural Biology, Weill Cornell Medicine, New York, New York, USA; 3 Department of Biochemistry, Weill Cornell Medicine, New York, New York, USA

**Keywords:** tau protein (tau), post-translational modification, NMR, acetylation, succinylation, phosphorylation, microtubule, lipid, Abl, abelson tyrosine kinase, AD, Alzheimer's disease, AF, amplification factor, BPS, phosphatidylserine isolated from porcine brain, D_2_O, deuterium oxide, ESR, electron spin resonance, HSQC, heteronuclear single quantum coherence, MBD, microtubule-binding domain, MT, microtubule, MW, molecular weight, PHF, paired helical filament, POPC, 1-palmitoyl-2-oleoyl-*sn*-glycero-3-phosphocholine, POPS, 1-palmitoyl-2-oleoyl-*sn*-glycero-3-phospho-l-serine, PTM, post-translational modification, STD, saturation transfer difference, SUV, small unilamellar vesicle, ThT, thioflavin T

## Abstract

Post-translationally modified tau is the primary component of tau neurofibrillary tangles, a pathological hallmark of Alzheimer's disease and other tauopathies. Post-translational modifications (PTMs) within the tau microtubule (MT)-binding domain (MBD), which encompasses two hexapeptide motifs that act as critical nucleating regions for tau aggregation, can potentially modulate tau aggregation as well as interactions with MTs and membranes. Here, we characterize the effects of a recently discovered tau PTM, lysine succinylation, on tau–tubulin interactions and compare these to the effects of two previously reported MBD modifications, lysine acetylation and tyrosine phosphorylation. As generation of site-specific PTMs in proteins is challenging, we used short synthetic peptides to quantify the effects on tubulin binding of three site-specific PTMs located within the PHF6^∗^ (paired helical filament [PHF] residues 275–280) and PHF6 (residues 306–311) hexapeptide motifs: K280 acetylation, Y310 phosphorylation, and K311 succinylation. We compared these effects to those observed for MBD PTM-mimetic point mutations K280Q, Y310E, and K311E. Finally, we evaluated the effects of these PTM-mimetic mutations on MBD membrane binding and membrane-induced fibril and oligomer formation. We found that all three PTMs perturb tau MT binding, with Y310 phosphorylation exerting the strongest effect. PTM-mimetic mutations partially recapitulated the effects of the PTMs on MT binding and also disrupted tau membrane binding and membrane-induced oligomer and fibril formation. These results imply that these PTMs, including the novel and Alzheimer's disease–specific succinylation of tau K311, may influence both the physiological and pathological interactions of tau and thus represent targets for therapeutic intervention.

Alzheimer's disease (AD) progresses in several stages, with tau neurofibrillary tangles and amyloid plaques accumulating in concert with cognitive impairment (1, 2, 3). Tau is a major contributor to neurodegeneration in a number of other diseases, collectively termed tauopathies (4, 5, 6, 7). Neurofibrillary tangles are composed of paired helical filaments and straight filaments and are associated with the neurodegeneration and brain atrophy that are observed at later stages of disease (1, 8). Tau pathology has been shown to spread across brain regions in a stereotypical manner (3), which has led to investigations of the mechanisms of tau spread. Soluble forms of tau, including post-translationally modified tau or mislocalized tau, often in oligomeric form, are commonly considered to be the toxic species in disease (5, 9). Evidence has also shown that it is possible for soluble forms of pathological tau to spread from cell to cell (9). Here, we investigate the ability of post-translational modifications (PTMs) and their mimetics within two paired helical filament (PHF) nucleating regions, PHF6^∗^ and PHF6, to alter tau interactions and the ability of tau to aggregate and thereby to contribute to tau's pathological role in forming toxic bodies.

Although the terminal stages of AD are recognized to encompass two pathological hallmarks, tau tangles and amyloid plaques, the onset and progression of earlier stages of disease are complex and remain poorly understood. Importantly, novel aspects of tau-mediated dysfunction in AD continue to be uncovered, including roles in disruption of functional hyperemia (10), neuroinflammation (11, 12), and metabolic deficits (13). While tau has long been known to be hyperphosphorylated in AD, a slew of additional tau PTMs have come to light, including acetylation and ubiquitination (14, 15, 16), as well as most recently, succinylation (13). Importantly, the role of such PTMs in disease mechanisms is not well understood, in part because their effects on the molecular interactions of tau remain to be fully characterized.

Here, we explore the effects of a novel modification of tau protein, lysine succinylation, on tau interactions and aggregation. Succinylation of proteins is common within mitochondria and contributes to sustaining proper metabolic function (17, 18, 19). Cellular metabolism is negatively impacted in AD and other dementias (20, 21). Altered cellular metabolism contributes to alterations in protein succinylation both within the mitochondria and in the cytosol (22, 23). Recent work indicates that these metabolic deficits result in site-specific tau succinylation in residue K311, located within the PHF6 hexapeptide motif (residues 306–311), a region that is strongly implicated in tau microtubule (MT) binding and aggregation (13). Succinylation of tau K311 was present in AD brains only, and never in control brains, suggesting a link to disease (13). We first examine the effects of K311 succinylation on tau binding to tubulin and compared them with those of previously reported modifications, acetylation of residue K280 and phosphorylation of residue Y310 that also occur within either the PHF6 motif or the corresponding PHF6^∗^ motif (residues 274–280).

K280 acetylation was characterized *in vitro* and shown to occur both in mouse models of AD and in human AD brains as well as in other tauopathies (24, 25, 26). Tau acetylation was reported to perturb MT assembly and promote tau aggregation (24). Site-specific acetylation of tau at residue K280, like succinylation of K311, was identified in AD/tauopathy brains only and not in control brains (25), suggesting an intimate link to disease. Importantly, the introduction of acetylation mimetics in the form of K280Q has been extensively used to assess and model the effects of K280 acetylation (24, 27, 28), but the fidelity of this mimetic to the authentic PTM has not been carefully assessed. Here, we show that the K280Q acetylation mimetic only partly recapitulates the effects of K280 acetylation on tau–tubulin interactions. While serine and threonine phosphorylation are well-established modifications of tau that have been shown to alter tau-MT binding and tau aggregation (29, 30, 31, 32), tau tyrosine phosphorylation has also begun to draw attention (33). Recently, the phosphorylation of residue Y310 by the abelson tyrosine kinase (c-Abl) has been shown to influence tau-mediated MT assembly and tau aggregation (33). Importantly, both tyrosine phosphorylation and c-Abl are of interest because of potential pathogenic roles in AD and other tauopathies (31, 33, 34, 35, 36, 37, 38), as well as other neurodegenerative diseases (39, 40).

The catalysts for tau aggregation *in vivo* remain unclear, but negatively charged molecules, including heparin, nucleic acids, and lipids, have all been shown to facilitate tau fibril formation *in vitro*. The tau MT-binding domain (MBD), which mediates tau–MT interactions (41, 42, 43, 44, 45, 46, 47, 48, 49) is commonly considered to play a critical role in tau aggregation (30, 50, 51, 52, 53, 54, 55). The MBD also mediates direct tau–lipid interactions (56, 57, 58, 59, 60, 61), and we have recently reported that these interactions can lead to the formation of tau oligomers with a nascent beta-strand character (53). Furthermore, recent structures of disease-associated tau fibrils extracted from human brains suggest the presence of cofactors with hydrophobic or negatively charged character (62, 63), consistent with the possibility that lipids may play a role in tau self-assembly in disease. We therefore also examined the effects of our three PTM-mimicking mutations on MBD membrane binding and membrane-induced oligomer and fibril formation. Because protein–membrane interactions are generally, though not always, less specific in nature than protein–protein interactions, we expect that the PTM mimetics may better represent the effects of the authentic PTMs in this context. Our results indicate that all three mutations reduce tau–membrane interactions, with the two mutations that introduce a negative charge having the stronger effect. All three mutations also alter the dimensions of membrane-induced tau oligomers, without altering the region involved in forming the core of the oligomers. Finally, all three mutations slowed membrane-induced tau fibril formation. Our results clarify the potential for PTMs situated within the tau PHF nucleating hexapeptide motifs to modulate functional and pathological tau interactions.

## Results

### Monitoring tau MBD–tubulin interactions using K18 and T2R

**Figure 1:**
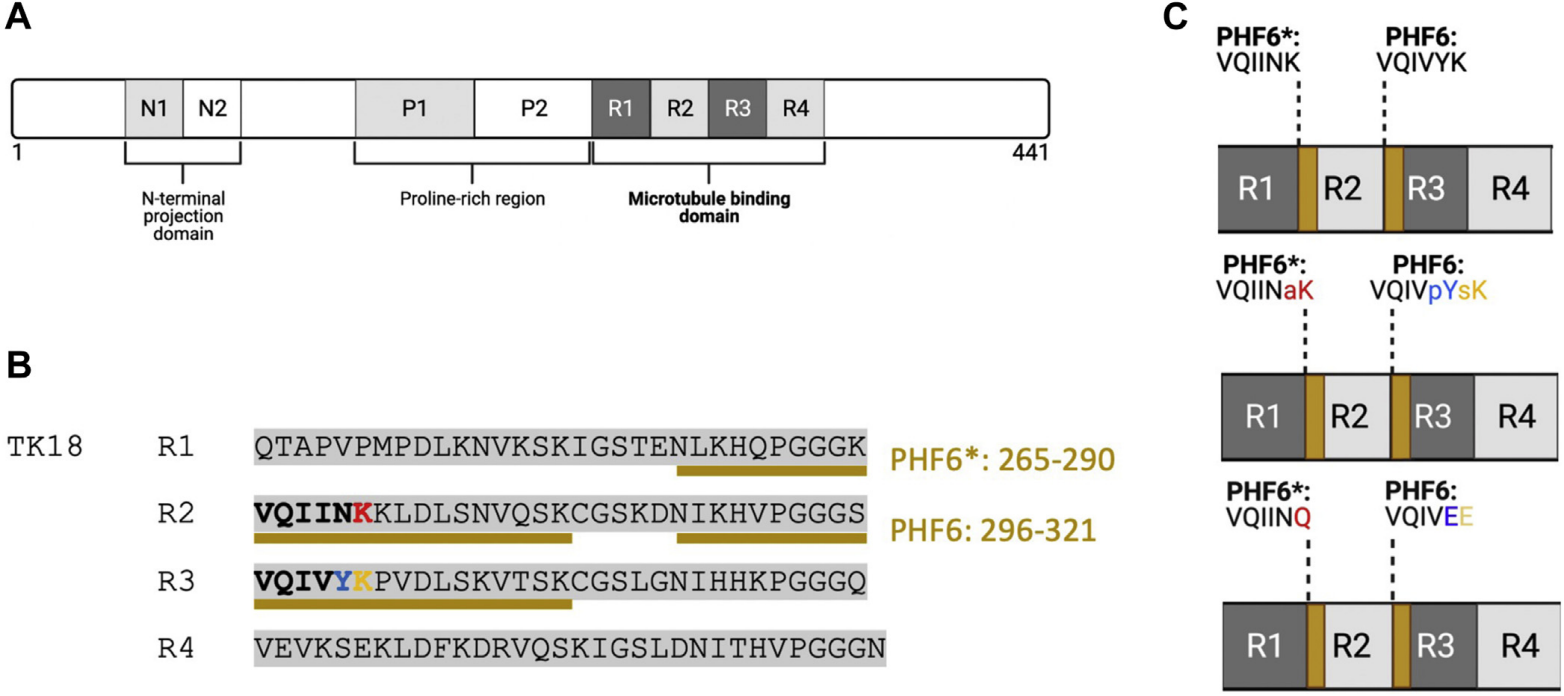
**Overview of tau and the constructs used in this study.** In its longest isoform (*A*), tau encompasses an N-terminal projection domain, a proline-rich region, and a microtubule-binding domain (MBD). For our studies, we used a tau construct known as K18 (*B*), comprised of residues 244 to 368. K18 includes all four MBD repeats and the PHF nucleating hexapeptide motifs, PHF6^∗^ and PHF6 (*boldface* and *C*, *upper panel*). We also used two synthetic peptides (*underlined* in *yellow*), each containing either the PHF6^∗^ or the PHF6 hexapeptide motifs as indicated in *yellow*. The three PTMs investigated in this study, K280 acetylation (*C*, “aK,” in *red*), Y310 phosphorylation (*C*, “pY,” in *blue*), and K311 succinylation (*C*, “sK,” *yellow*) are indicated (*C*, *middle panel*) as well as the three point mutants (K310Q, Y310E, and K311E) used to mimic these PTMs (*C*, *lower panel*). PHF, paired helical filament; PTM, post-translational modification.

Tau is composed of 441 amino acids comprising the N-terminal projection domain, proline-rich domain, and MBD (Fig. 1*A*). Different isoforms of tau are present in the brain and occur because of alternative splicing of exons 2 and 3, or exon 10, which determine the presence of the first or second N-terminal domain and the second repeat region within the MBD. The MBD is directly involved both in PHF formation, tau–MT interactions, and tau–membrane interactions (64). The MBD contains four imperfect 30 to 31 residue repeats (R1, R2, R3, and R4). Within MBD repeats R2 and R3, there are two hexapeptide motifs known as PHF6^∗^ and PHF6 (Fig. 1*B*) that are important nucleating regions for filament formation (50, 51, 65). PTMs within the PHF6∗ and PHF6 motifs may therefore influence both tau function and its pathological role in AD. Because of the importance of the MBD for both tau function and tau self-assembly, we chose to work with the commonly used MBD construct K18 (49, 52, 58, 66) (Fig. 1*B*).

**Figure 2:**
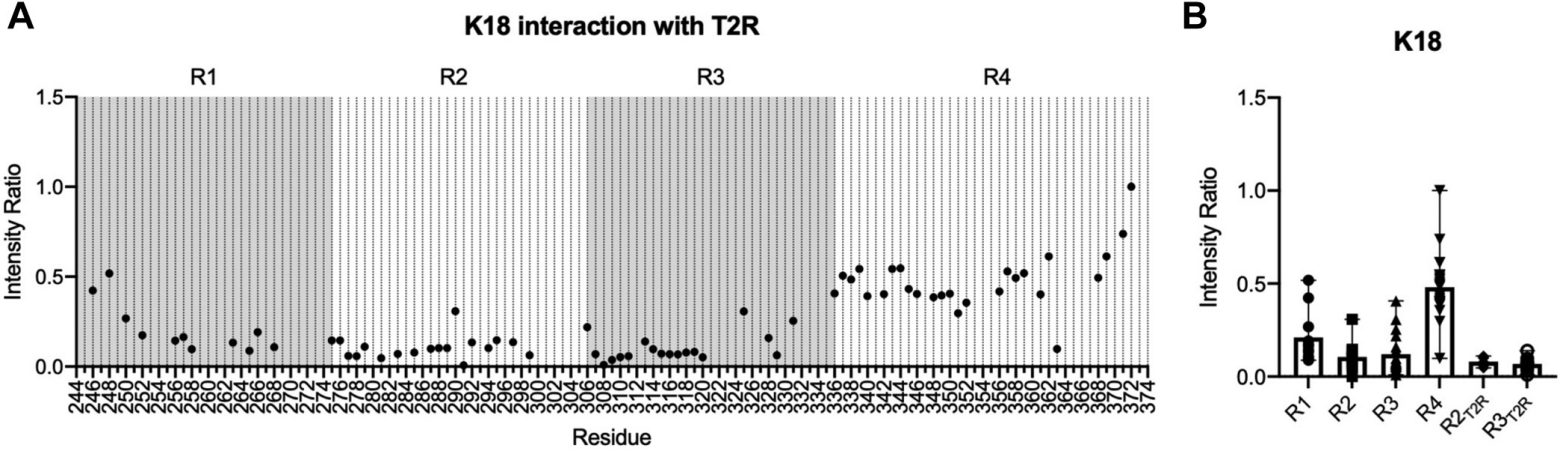
**Identifying**^**15**^**N-K18 residues and core regions R2**_**T2R**_**and R3**_**T2R**_**that interact with T2R.***A,*^1^H,^15^N HSQC spectra (Fig. S4*A*) were recorded for K18 (42 μM) in the absence and presence of T2R (50 μM). Intensity ratios were calculated for assigned resolved resonances, based on previous assignments (58, 97), as the intensity in the presence of T2R normalized by the corresponding intensity in the absence of T2R. MBD repeats are indicated in alternating *gray shading*. *B*, intensity ratios of residues were grouped and averaged based on their corresponding location within repeat regions (R1–R4). Intensity ratios were also averaged over regions we define as R2_T2R_ (residues 277–289) and R3_T2R_ (residues 308–320), identified as exhibiting the lowest intensity ratios and therefore the tightest binding to T2R. Data shown in (*B*) represent the mean (*bar*) and range (*whisker*) for all resolved resonances within each region, with the individual intensity ratios indicated as symbols. HSQC, heteronuclear single quantum coherence; MBD, microtubule-binding domain.

We modeled tau–tubulin binding by using the T2R complex composed of two tubulin heterodimers stabilized by the stathmin-like domain RB3 (67, 68). T2R is a rigid stable complex that avoids spontaneous polymerization/depolymerization of tubulin and presents an elongated surface that resembles that of an assembled MT. The binding of tau K18 to T2R was previously characterized using NMR spectroscopy to quantify intensity ratios for individual residues (44). Intensity ratios are determined as the intensity values in ^15^N–^1^H heteronuclear single quantum coherence (HSQC) NMR spectra for well-resolved resonances of tau K18 in the presence of T2R (bound state) normalized by the intensity values for the same residues in the absence of T2R complex (free state). Evidence of binding can be visualized by decreased resonance intensities in the spectra with T2R, resulting in intensity ratios lower than 1. For many resonances, intensities in the presence of T2R were nearly undetectable (Fig. S4*A*), resulting in intensity ratios near zero (Fig. 2*A*). Our results reproduce the previously observed intensity ratio pattern of WT K18 in the presence of T2R and indicate strong binding of K18 between residues 258 and 320 (44), although we report data for a greater number of signals. To facilitate comparison of WT K18 to other variants, we grouped and averaged intensity ratios according to repeat boundaries of the MBD (R1, R2, R3, and R4). Plotted in this manner, the data show that the R2 and R3 repeats bind more tightly to T2R in comparison to R1 and R4, which have higher average intensity ratio values (Fig. 2*B*). The lowest intensity ratios within K18, indicative of the regions with strongest binding to T2R, are comprised of residues 277 to 289 and 308 to 320. We separately averaged the intensity ratios within these two regions and refer to them as core T2R-binding regions R2_T2R_ (277–289) and R3_T2R_ (308–320) (Fig. 2*B*).

### Effects of succinylation, acetylation, and tyrosine phosphorylation on MBD–tubulin interactions

To generate succinylated and acetylated samples, we incubated K18 with succinyl-CoA or acetyl-CoA at a molar ratio of 1:28 (protein:acyl-CoA) stoichiometry at 37 °C overnight. To monitor PTM, we monitored lysine side-chain resonances in NMR HSQC spectra during the course of the reaction. Both succinylation and acetylation occur at the lysine epsilon amino site, and modification results in a shift of the modified epsilon amino resonance in the ^1^H and ^15^N dimension (69, 70) (Fig. S1). To assess the extent of either modification, we estimated the number of modified lysines by integrating the shifted lysine epsilon amino resonance and normalizing its volume by that of the backbone lysine NH resonance for a well-resolved lysine residue, K311, as previously reported (71), and normalized this value by the total number of lysines in K18. While incubation with succinyl-CoA resulted in a considerable extent of lysine succinylation (ca. 39.7% of the 20 K18 lysines), incubation with acetyl-CoA resulted in very little modification (ca. 1.4% of the 20 K18 lysines) (Table 1). However, we noted that the latter number may be an underestimate of the extent of acetylation, since at 37 °C proton exchange may diminish the signal from the acetyl-lysine epsilon amino groups, which have also been reported to exchange more rapidly than backbone protons (71). Tau K18 contains only a single tyrosine residue, Y310, which was phosphorylated *in vitro* using recombinant c-Abl kinase. Phosphorylation can be monitored by NMR *via* shifts in resonances for residues close to the site of modification, including V309, Y310, and K311 (33). As previously reported, we could achieve quantitative phosphorylation of Y310 using this approach (33).

**Table 1:** Extent of modification by nonenzymatic succinylation and nonenzymatic acetylation as measured by NMR

Construct	Peak	Volume	^15^N ppm	^1^H ppm	% Modified
Acetyl-K18	N ε-peak	19.5	127.5	8.0	1.4
	K311 peak	68.8	126.4	8.2	
Succinyl-K18	N ε-peak	356.5	125.7	7.9	39.7
	K311 peak	44.9	126.3	8.2	

The number of modified lysines was obtained by integrating the shifted lysine epsilon amino resonance and normalizing its volume by that of the backbone lysine NH resonance for a well-resolved lysine residue, K311, as previously reported (71) and was normalized by the total number of lysines (20) in K18. Peak volumes and positions of the corresponding modified epsilon amino resonance and the unmodified K311 peak are shown based on ^1^H,^15^N HSQC NMR spectra taken at the end of each reaction (nonenzymatic acetylation or succinylation).

**Figure 3:**
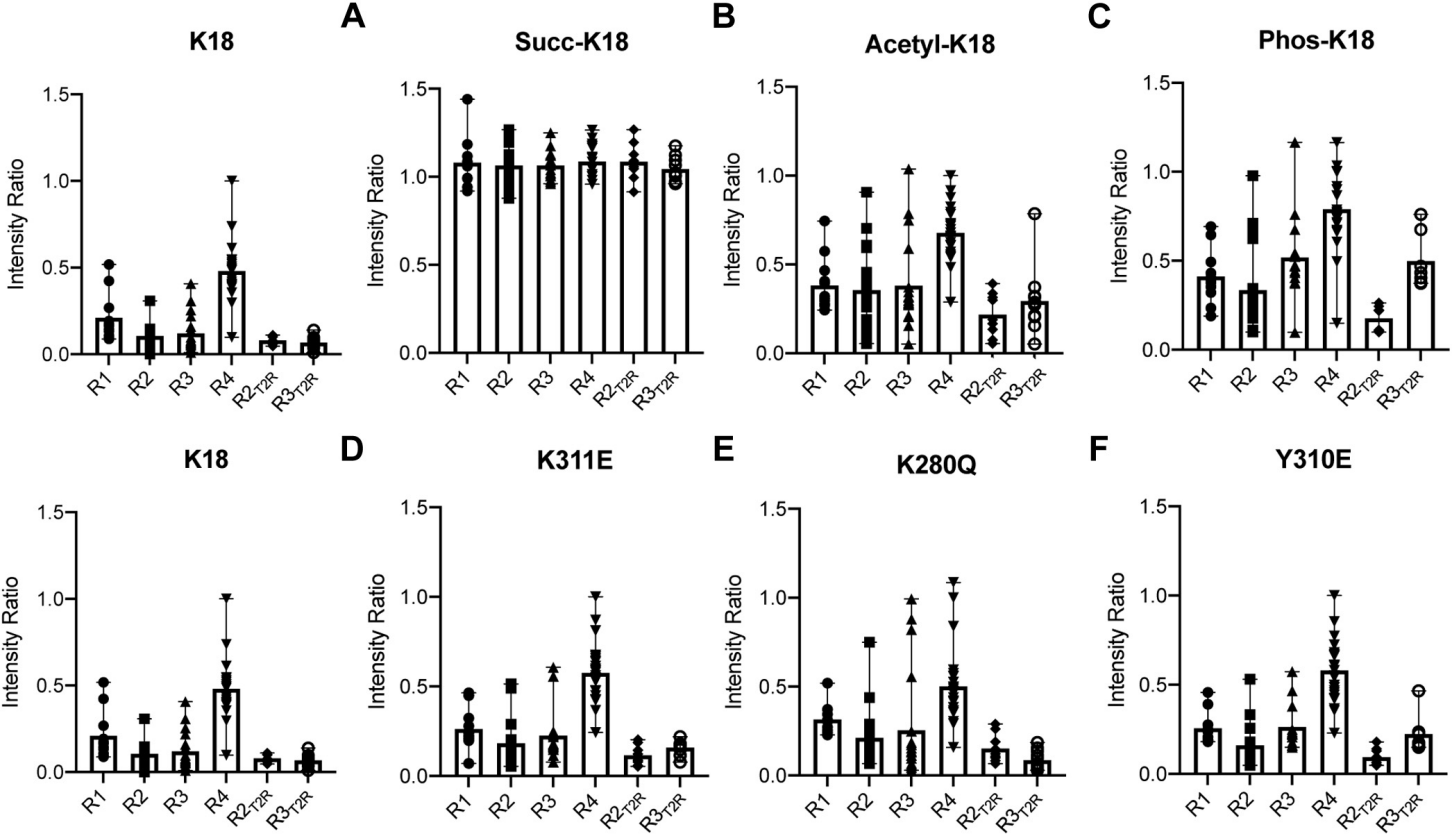
**PTMs and PTM mimetics alter K18 binding to T2R.** Intensity ratios were calculated from ^1^H,^15^N HSQC spectra of samples (42 μM) with and without T2R (50 μM) and averaged over each MBD repeat as well as the core T2R-binding regions R2_T2R_ and R3_T2R_ for (*A*) succinylated K18, (*B*) acetylated K18, (*C*) phosphorylated K18, (*D*) succinylation mimetic K311E, (*E*) acetylation mimetic K280Q, and (*F*) phosphorylation mimetic Y310E. Data to the *left* of each row of panels are for unmodified K18 (same as Fig. 2*B*) and shown for ease of comparison. Data shown as mean (*bar*) and range (*whisker*) of all resolved resonances within each region with individual data points indicated as symbols. HSQC, heteronuclear single quantum coherence; MBD, microtubule-binding domain; PTM, post-translational modification.

To determine the effects of these PTMs on K18 binding to the T2R complex, we measured intensity ratios as described previously for unmodified K18. Succinylation of K18 nearly eliminates binding of K18 to T2R, as indicated by an increase in the intensity ratios to values near one throughout the MBD (Figs. 3*A* and S2*B*). Despite the limited degree of lysine acetylation that was achieved *via* incubation with acetyl-CoA, K18 prepared using this protocol exhibited decreased binding to T2R, as indicated by increased intensity ratios in all four MBD repeats, as well as in the R2_T2R_ and R3_T2R_ motifs (Figs. 3*B* and S2*C*). The magnitude of this effect also suggests that our estimate of the extent of lysine acetylation in our samples may be too low. Finally, K18 phosphorylated at Y310 also exhibited decreased binding to T2R, with increased intensity ratios throughout the protein, but with more pronounced effects in the R3 repeat and the R3_T2R_ motif (Figs. 3*C* and S2*D*).

### PTMs locally disrupt binding of tau to T2R

Nonenzymatic lysine succinylation and acetylation is relatively nonspecific, limiting our ability to assess the effect of site-specific succinylation of K311 or acetylation of K280 on tau–tubulin binding. Because generating isotopically labeled site-specifically succinylated or acetylated K18 in sufficient quantities for NMR remains challenging, we chose to examine the effects of PTMs on tubulin binding using short synthetic peptides, which can be obtained more readily. We chose peptides composed of residues 265 to 290, which encompasses the R2_T2R_ motif, and 296 to 321, which encompasses the R3_T2R_ motif, as both these peptides have previously been shown to bind with specificity to tubulin and MTs *in vitro* using saturation transfer difference (STD) NMR, whereas other tau peptides of similar length did not exhibit binding (41, 42). We obtained both unmodified and modified (succinyl-K311, acetyl-K280, and phospho-Y310) versions of the peptides and used STD NMR to monitor their binding to the T2R complex. The presence of an STD signal is directly associated with the presence of ligand–receptor interactions, where here the tau peptides represent the ligand and T2R the receptor.

As previously reported, we measured a robust STD signal throughout the aromatic and amide spectral regions for tau peptide 296 to 321, indicating binding to T2R (Fig. S3*A*), which included resonances previously assigned to the HD1 and HE1 protons of Y310 (41, 42). Introducing phosphorylation at residue Y310 results in a weaker STD signal (Fig. S3*B*). Similarly, loss of binding is evident when succinylation at residue K311 is introduced (Fig. S3*C*). For the 265 to 290 peptide, the STD signal is weaker, in part because of the absence of a tyrosine residue and its associated strong signals in the position equivalent to Y310. Nevertheless, the observed signal is further weakened by introducing acetylation at K280 (Fig. S3, *D* and *E*). These results indicate that MBD interactions with T2R are decreased by all three PTMs.

**Figure 4:**
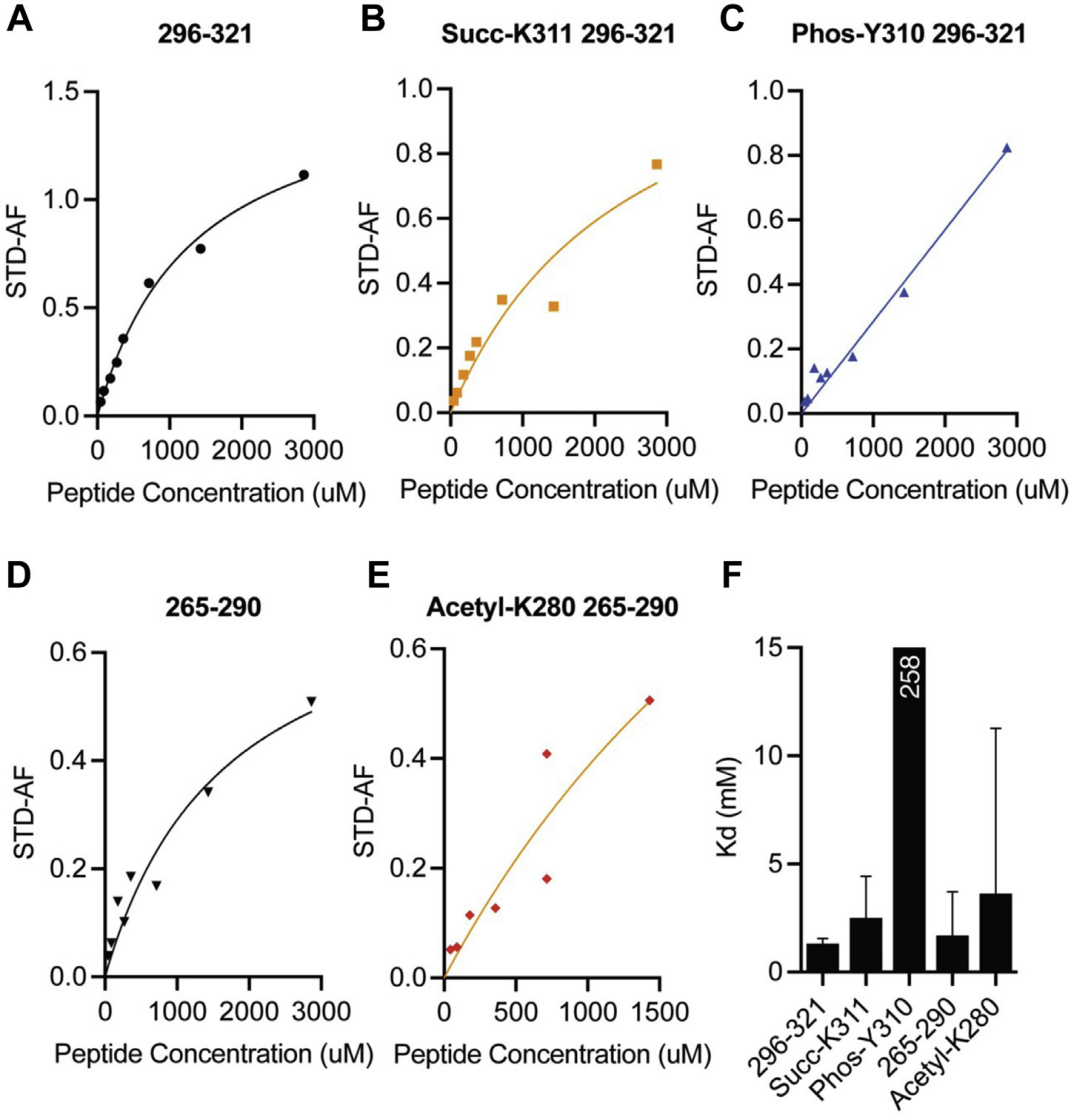
**Site-specific PTMs alter binding of tau peptides to T2R.** STD spectra were recorded and used to calculate STD_AF_ values in the presence of T2R for an unmodified synthetic peptide spanning tau residues 296 to 321 (*A*) and K311 succinylated (*B*) and Y310 phosphorylated (*C*) forms of the peptide, and for an unmodified synthetic peptide spanning tau residues 265 to 290 (*D*) and a K280 acetylated form of the peptide (*E*). Peptide concentrations ranged from 0.044 to 2.8 mM. Data were fitted to Equation 2 (see the Experimental procedures section) to determine apparent *K*_*d*_s (*F*). Weak binding for Y310 phosphorylated peptides precluded a reliable fit. Tau peptide concentrations ranged from 0.044 to 2.8 mM, and T2R concentration was 10 μM. AF, amplification factor; PTM, post-translational modification; STD, post-translational modification.

We attempted to assess the disruption of binding by PTMs more quantitatively using measurements of the STD amplification factor (AF) for a series of peptide concentrations (Fig. 4, *A*–*E*) to determine apparent *K*_*d*_ values (Fig. 4*F*) (72) (see the Experimental procedures section). The low affinities, combined with the limited solubility of the peptides, resulted in large errors for the estimated *K*_*d*_ values, and our interpretation is therefore qualitative rather than quantitative. Affinities for the unmodified peptides were in the low millimolar range, similar to measurements performed recently for a C-terminal tau peptide (72). The affinity of the 265 to 290 peptide, which is derived mostly from repeat R2, was similar to or slightly lower (higher apparent *K*_*d*_) than that of the 296 to 321 peptide, which is derived mostly from R3, consistent with an early study of the contributions of R2 and R3 to the affinity of the MBD for MTs (73). Succinylation at K311 resulted in a trend toward lower apparent affinity (ca. twofold increased *K*_*d*_) than that of the 296 to 321 peptide for T2R, and acetylation of K280 resulted in a similar trend toward lower affinity than that of the 265 to 290 peptide. In contrast, phosphorylation at Y310 resulted in a linear binding curve over the accessible range of peptide concentrations, precluding a robust fit to the data, but indicating a more severe reduction in affinity. These trends in the apparent *K*_*d*_ values are consistent with the decreases in the STD spectra observed for the three PTMs (Fig. S3).

### PTM mimetics partly reproduce PTM effects on T2R binding

**Figure 5:**
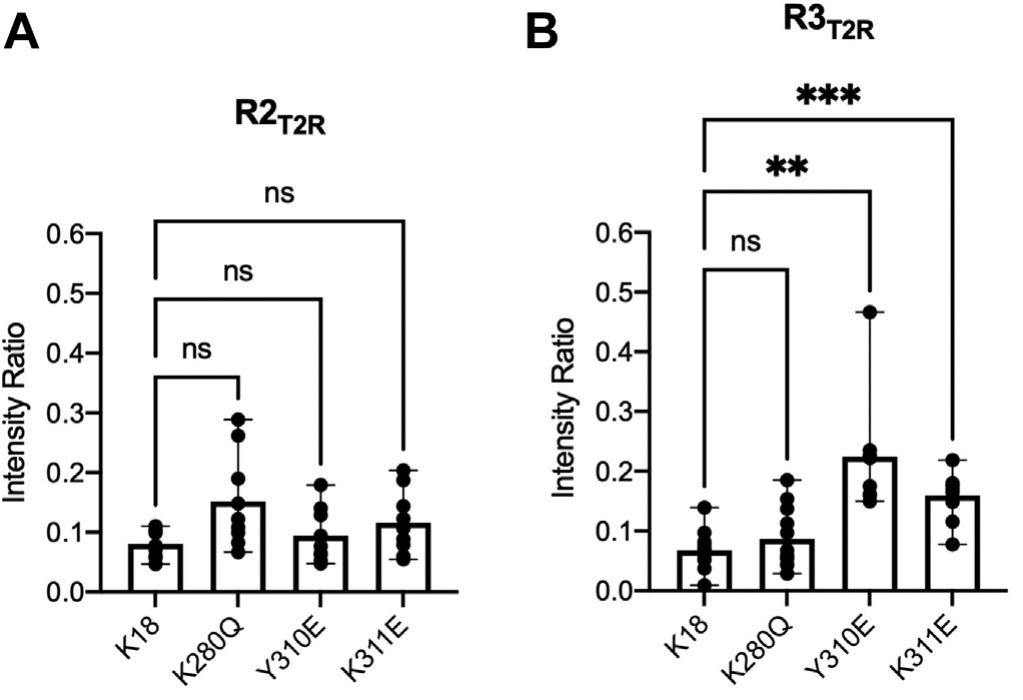
**PTM mimetics alter binding of R2**_**T2R**_**and R3**_**T2R**_**to T2R.***A* and *B*, R2_T2R_ and R3_T2R_ regions within K18 exhibit extensive binding to T2R. Loss of binding within both regions is observed for each PTM mimetic (42 μM) as indicated by an increase in mean intensity ratio in the presence of T2R (50 μM). The K280Q mutant exhibited decreased binding (higher average intensity ratio) in the R2_T2R_ region, though the difference did not reach statistical significance (*p* = 0.09). *B*, both the phosphorylation mimetic Y310E and the succinylation mimetic K311E decreased binding of the R3_T2R_ region. Statistical significance was assessed using one-way ANOVA followed by multiple-comparisons test of matched residues (∗*p* < 0.05, ∗∗*p* < 0.01, and ∗∗∗*p* < 0.001). PTM, post-translational modification.

A widely used approach for studying the effects of site-specific PTMs on protein function is the use of PTM-mimicking point mutations. Acetylation is typically mimicked using a glutamine substitution (24), succinylation using a glutamate (74, 75, 76, 77), and phosphorylation using an aspartate or a glutamate (39). To evaluate this strategy, we assessed the effects of PTM mimetics K280Q, Y310E, and K311E on the interactions of K18 with T2R and compared them to those observed for nonspecific succinylation and acetylation and authentic phosphorylation of K18 and for authentic modification of short peptides. We acquired ^15^N–^1^H HSQC spectra of each of the three mutants in the absence and presence of T2R (Fig. S4) and from these computed intensity ratios for well-resolved resonances (Fig. S5). We grouped and averaged intensity ratios as described previously to facilitate comparisons with unmodified K18 and with the effects of PTMs. We first examined the effects of the mutations on the core R2_T2R_ and R3_T2R_ tubulin-binding regions, in which the mutations and PTM sites of interest reside (Fig. 5). The acetylation mimetic K280Q resulted in decreased binding for R2_T2R_ motif compared with unmodified K18 (Fig. 5*A*), although the difference did not reach statistical significance (*p* = 0.092) but did not affect the binding of the R3_T2R_ motif (Fig. 5*B*). Both the Y310E phosphomimetic and the K311E succinylation mimetic decreased binding of the R3_T2R_ motif without affecting the R2_T2R_ motif, with Y310E showing a stronger effect (Fig. 5). We next compared the effects of the mutations to our measurements of succinylated, acetylated, or phosphorylated K18 across all four repeats and the two T2R-binding motifs (Figs. 3 and S6). The effects of K311E (Figs. 3*D* and S6*A*, *green data*) were much smaller and more localized than for nonspecific succinylation of K18 by incubation with succinyl-CoA (Figs. 3*A* and S6*A*, *red data*), which is not surprising given that approximately eight lysines are succinylated by the latter method, likely perturbing binding at multiple sites. Similarly, the effects of K280Q (Figs. 3*E* and S6*B*) were less than those of incubation with acetyl-CoA, despite the limited extent of lysine acetylation achieved using our protocol. Finally, the effects of Y310E (Figs. 3*F* and S6*C*) were similar to those of Y310 phosphorylation but lesser in magnitude.

### MBD–lipid binding is mediated by both amphipathic helices and hexapeptide motifs

**Figure 6:**
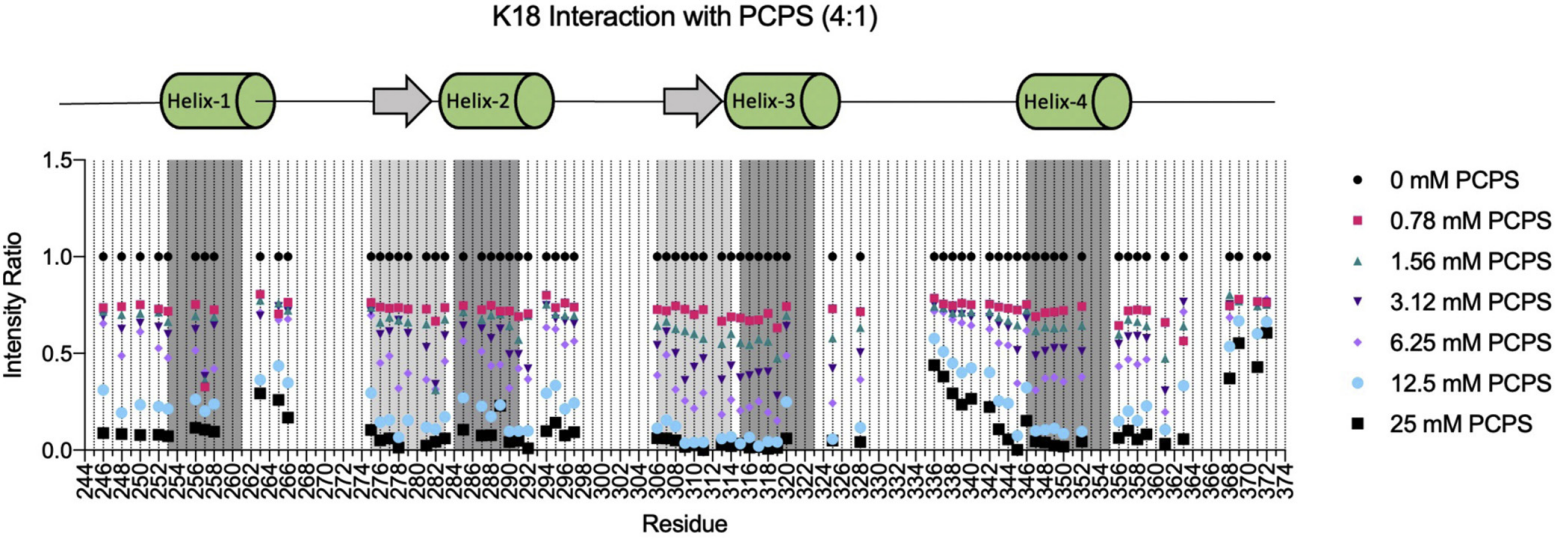
**Lipid-binding sites of tau K18 include amphipathic helices and hexapeptide motifs.** Intensity ratios were calculated from ^1^H,^15^N HSQC spectra of tau K18 (62.5 μM) in the presence of increasing concentrations (0–25 mM) of 4:1 POPC:POPS SUVs. Regions corresponding to previously characterized amphipathic helices (helices 1–4) shown to mediate lipid interactions are highlighted in *dark gray* and by *green cylinders* above the plot. The N-terminal regions of R2 and R3, denoted PHF6^∗^+ and PHF6+, which also exhibit strong binding and include the PHF6^∗^ and PHF6 hexapeptide motifs, are highlighted in *light gray* and indicated by *arrows* above the plot. HSQC, heteronuclear single quantum coherence; PHF, paired helical filament; POPC, 1-palmitoyl-2-oleoyl-*sn*-glycero-3-phosphocholine; POPS, 1-palmitoyl-2-oleoyl-*sn*-glycero-3-phospho-l-serine; SUV, small unilamellar vesicle.

The tau MBD can bind directly to lipid bilayers, interacting with membranes in part *via* short amphipathic helices formed within each of the MBD repeats (56, 57). While the physiological significance of tau–membrane interactions remains unclear, a number of studies have demonstrated that lipid binding can facilitate tau self-assembly into fibrils and/or oligomers (33, 78, 79, 80, 81). We therefore examined the effects of PTM-mimetic mutations on K18 binding to lipid vesicles. Because we previously showed that vesicles that are high in negatively charged lipid content efficiently drive tau aggregation (53), we used small unilamellar vesicles (SUVs) composed of 4:1 1-palmitoyl-2-oleoyl-*sn*-glycero-3-phosphocholine (POPC) to 1-palmitoyl-2-oleoyl-*sn*-glycero-3-phospho-l-serine (POPS). ^15^N–^1^H HSQC NMR spectra of K18 in the presence of increasing concentrations of SUVs resulted in intensity losses in many signals, which we quantified as intensity ratios for signals in the presence *versus* absence of SUVs (Fig. 6). As expected, based on our previous studies of tau–micelle (58) and tau–membrane (57) interactions, the previously annotated amphipathic helix motif within each repeat (helix 1: residues 253–261, helix 2: 284–291, helix 3: 315–323, and helix 4: 346–355) exhibited strong interactions with the vesicles, as indicated by their lower intensity ratios at intermediate lipid concentrations. In addition, N-terminal regions to the helical motifs in R2 and R3, including the PHF6 and PHF6^∗^ motifs, bound to vesicles as strongly as the helical region at the different lipid concentrations. Notably, these regions also exhibited restricted mobility in the presence of micelles (58), suggesting they may interact with lipids, but direct observations of membrane interactions of the tau MBD hexapeptide motifs have not been previously reported.

**Figure 7:**
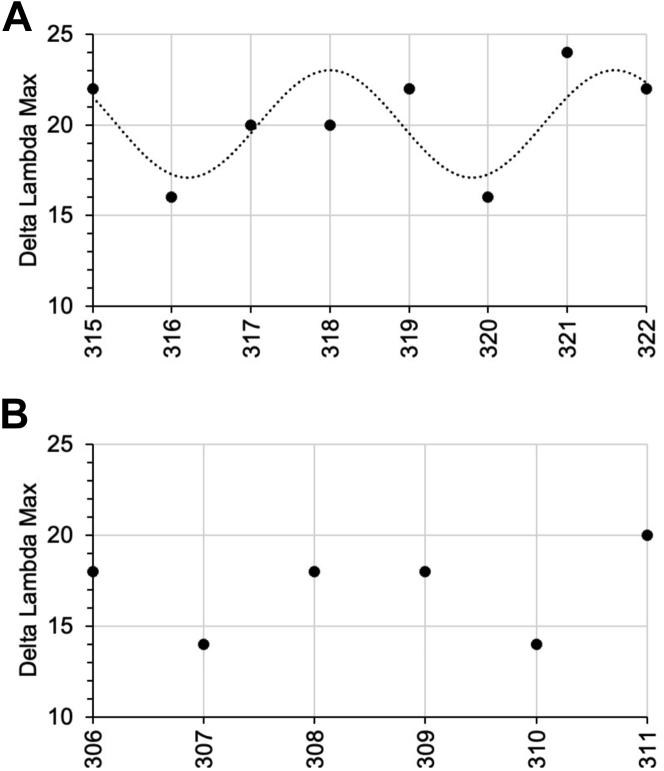
**Tryptophan fluorescence reveals binding of tau helix-3 and PHF6 regions to membranes.** Binding of tau fragment K16 (20 μM) with Trp residues introduced at residues (*A*) 315 to 322 and (*B*) 306 to 311 in the presence of 4:1 POPC:POPS SUVs (30 mM). *Symbols* indicate the calculated delta lambda max, and *dashed line* in (*A*) indicates the curve fitted to a periodic function with a periodicity of 3.6 (see the Experimental procedures section) for residues 315 to 322. Data in *A* are taken from Ref. (59) but are fit differently here. PHF, paired helical filament; POPC, paired helical filament; POPS, 1-palmitoyl-2-oleoyl-*sn*-glycero-3-phospho-l-serine; SUV, small unilamellar vesicle.

We previously monitored changes in electron spin resonance (ESR) spectra of environmentally sensitive spin labels to establish a helical periodicity in the amphipathic helix regions in the presence of vesicles (57). To assess and confirm the interactions of PHF6 region with membranes, we recently adapted this method to allow for more rapid fluorescence measurements by generating a series of Trp mutants and measuring changes in their fluorescence spectra in the presence and absence of 1:1 POPC:POPS SUVs (59). We first examined the helix 3 region to determine whether this approach was consistent with our previous ESR measurements (57). Changes in the wavelength of maximal fluorescence (delta lambda max) for Trp mutants spanning residues 315 to 322 exhibited a helical periodicity (Fig. 7*A*), as previously observed using ESR. Measurements using single cysteine mutants labeled with the fluorophore acrylodan gave similar results (Fig. S7), alleviating potential concerns regarding the effect of the specific modification used. Having established the validity of this approach, we then examined Trp mutants within the PHF6 hexapeptide motif. Trp mutants spanning the PHF6 motif also showed clear changes in delta lambda max in the presence of vesicles (Fig. 7*B*), confirming a direct interaction of this region with the membrane surface. Although no clear periodicity associated with regular secondary structure could be established for this short stretch and hydrophobic or positively charged residues showed the largest changes, consistent with hydrophobic and electrostatic interactions of these residues with the negatively charged lipid membrane.

### PTM mimetics disrupt MBD binding to lipid membranes

**Figure 8:**
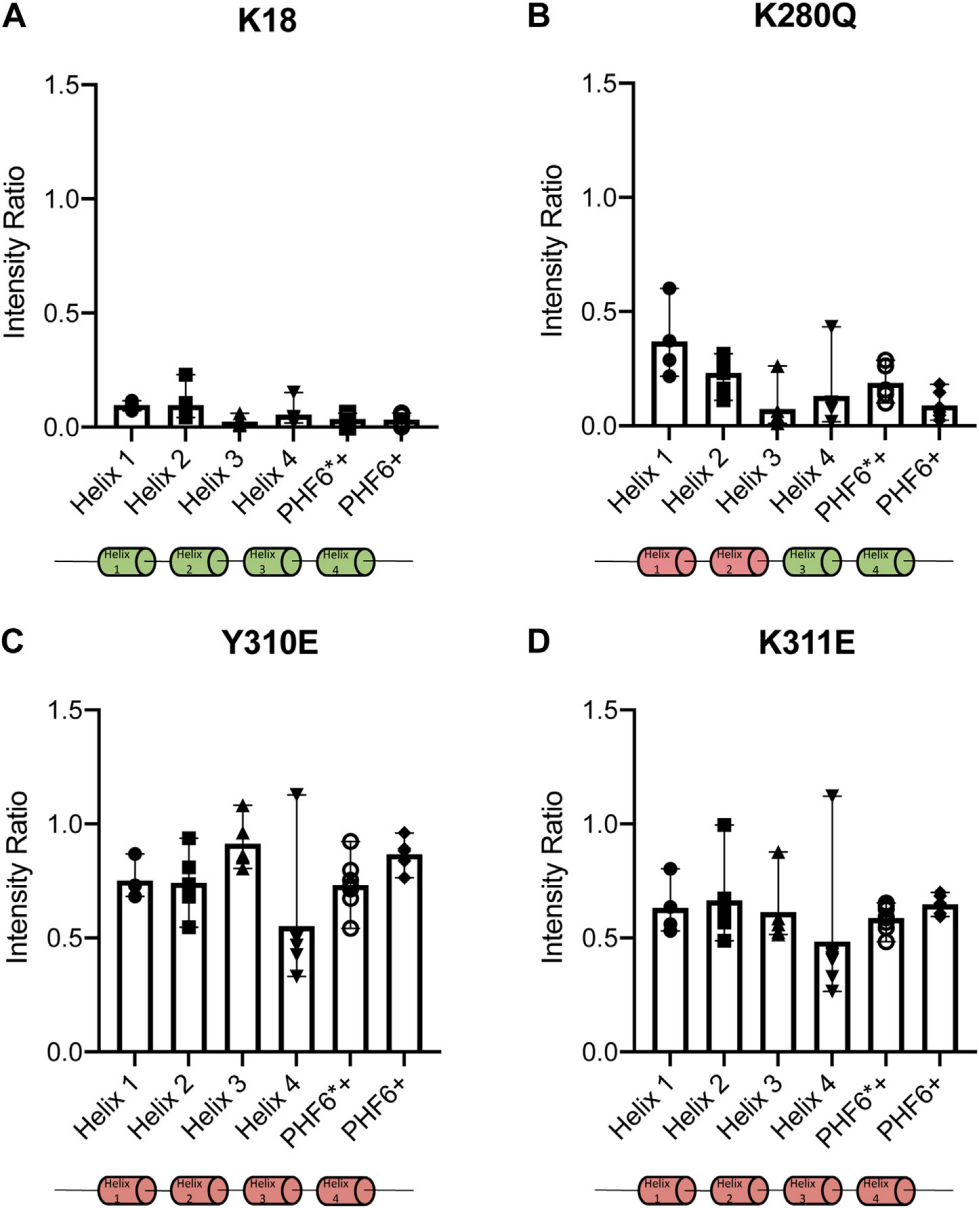
**PTM mimetic mutations within lipid-binding regions PHF6^∗^+ and PHF6+ alter binding of the MBD to lipids.** Intensity ratios of K18 (62.5 μM) with or without 25 mM 4:1 POPC:POPS SUVs (25 mM) were averaged over regions corresponding to amphipathic helices (helices 1–4) and the PHF6^∗^+ and PHF6+ regions. For unmodified construct K18 (*A*), and PTM mimetics K18 K280Q (*B*), K18 Y310E (*C*), and K18 K311E (*D*). Data shown are mean (*bar*) and range (*whisker*) for resolved signals within each region, with individual intensity ratios indicated as symbols. *Cartoon* under each plot indicates helical regions of K18 that bind to (*green*) or exhibit perturbed binding to (*red*) lipids. MBD, microtubule-binding domain; PHF, paired helical filament; POPC, 1-palmitoyl-2-oleoyl-*sn*-glycero-3-phosphocholine; POPS, 1-palmitoyl-2-oleoyl-*sn*-glycero-3-phospho-l-serine; PTM, post-translational modification; SUV, small unilamellar vesicle.

We then examined the effects of the three PTM-mimetic mutations on K18–vesicle binding intensity ratios. To facilitate comparison with the unmodified protein, we averaged the intensity ratios over each of the four amphipathic helix regions (helices 1–4) as well as over the R2 and R3 N-terminal hexapeptide-containing regions, which we define as residues 275 to 283 (termed PHF6^∗^+) and 306 to 314 (termed PHF6+) (Fig. 8*A*). The acetylation mimetic K280Q decreased binding of helix 1, helix 2, and the PHF6^∗^+ motif located in R2 but did not affect binding of helix 3, helix 4, or the PHF6+ motif in R3, indicating a relatively local perturbation of membrane affinity (Figs. 8*B* and S8*B*). In contrast, both the succinylation mimetic K311E (Figs. 8*C* and S8*C*) and the phosphorylation mimetic Y310E (Figs. 8*D* and S8*D*) lead to globally decreased binding in all four helices as well as both hexapeptide-containing regions, with Y310E exerting a slightly stronger effect.

### PTM mimetics perturb membrane-induced tau oligomer formation

**Figure 9:**
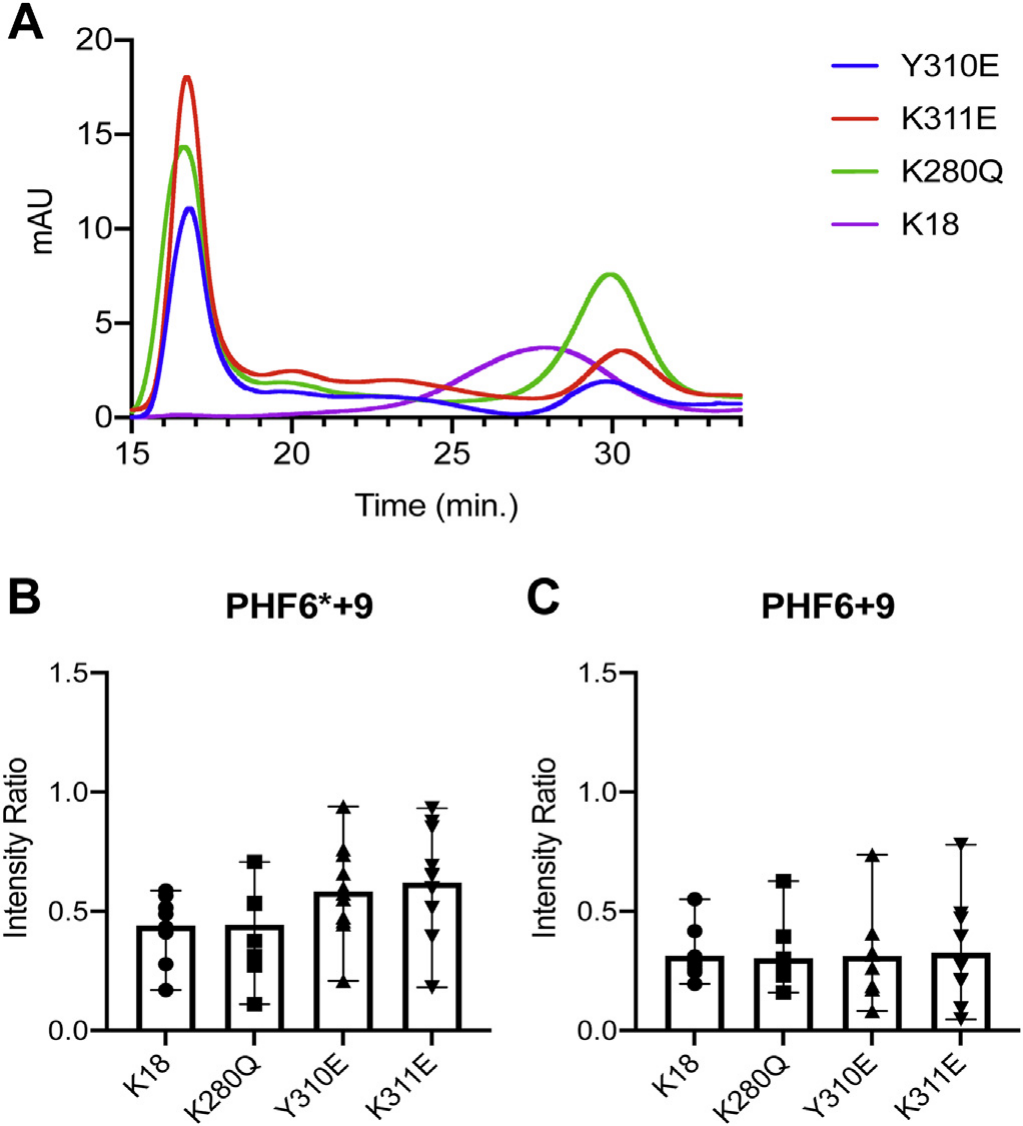
**PTM mimetics delay elution of membrane-induced tau oligomers but do not alter the oligomer core.** Oligomers were induced by incubation of unmodified and mutant K18 (200 μM) with BPS vesicles (4 mM) for 24 h at 37 °C. *A*, SEC profiles of oligomers prepared from unmodified and mutant K18. Oligomers elute at 28 min for the unmodified protein, as previously reported (53) and confirmed by SDS-PAGE (not shown). Oligomers elute somewhat later for the three PTM-mimetic mutants. Unconverted lipid vesicles elute in the void volume. *B* and *C*, intensity ratios (I/I_0_) of residues previously shown to comprise the oligomer core (53) were calculated from ^1^H,^15^N HSQC spectra of oligomeric complexes and monomeric tau and averaged over the PHF6^∗^+9 (*B*) or PHF6+9 (*C*) regions. BPS, phosphatidylserine isolated from porcine brain; HSQC, heteronuclear single quantum coherence; PHF, paired helical filament; PTM, post-translational modification; SEC, size-exclusion chromatography.

We previously reported that tau K18, as well as full-length tau, form oligomeric protein–lipid complexes upon incubation with lipid vesicles, with conversion being most efficient at higher contents of negatively charged lipids (53). We examined whether the loss of membrane binding observed previously for the three PTM mimetics alters the formation of tau–phospholipid oligomeric complexes, as we previously showed that a membrane-binding–deficient quadruple mutant eliminated oligomer formation (53). We first monitored oligomer formation using size-exclusion chromatography. We found that each of the three PTM mimetics remained capable of forming membrane-induced K18 oligomers but delayed the elution time of the oligomers when compared with that of unmodified K18 oligomers (Fig. 9*A*). Furthermore, oligomer formation for the three mutants was less efficient than for the unmodified protein, as indicated by the large void peak observed for the mutants, which contains intact lipid vesicles that were not converted to oligomers. Oligomer formation for all three mutants was also confirmed using negative-stain electron microscopy (Fig. S9).

Delays in oligomer elution time could result from changes in molecular weight (MW) or from conformational changes. To assess if the changes observed resulted from changes in the residues that comprise the oligomeric core, which might be expected to alter oligomer conformation, we used solution-state NMR to quantify the intensity ratios of oligomeric preparations for each mutant (Fig. S10). We previously showed using both solid-state and solution NMR that the core of K18 oligomers core is composed of the two hexapeptide motifs, PHF6^∗^ and PHF6, as well as of ca. nine residues subsequent to each motif (53). For these two regions, here termed PHF6^∗^+9 and PHF6+9, we averaged intensity ratios calculated from spectra of oligomer preparations normalized by spectra of free protein (Fig. 9, *B* and *C*). Our results indicate that the oligomeric core is largely conserved in the three PTM mimetics, and that changes in the regions comprising the oligomer core are not responsible for the shifts observed in the elution time of oligomers formed using the PTM-mimetic mutations.

### PTM mimetics perturb membrane-facilitated tau fibril formation

**Figure 10:**
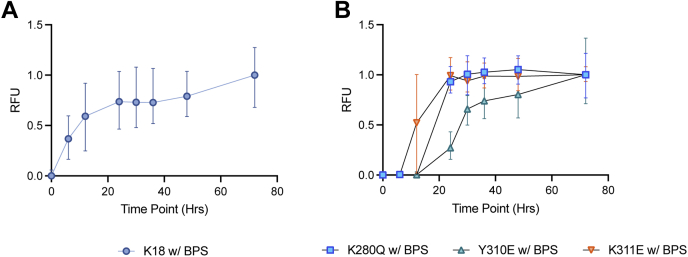
**PTM mimetics alter membrane-induced fibril formation.** Unmodified (*A*) and mutant (*B*) K18 at 200 μM concentration was incubated with 4 mM BPS vesicles while shaking at 1000 RPM, and normalized ThT fluorescence was used to monitor fibril formation. *T*_50_ values were calculated as the time to reach half-maximal fluorescence and are tabulated in Table 2. Data shown are mean and range of three independent experiments, normalized to the final data point at 72 h. BPS, phosphatidylserine isolated from porcine brain; PTM, post-translational modification; ThT, thioflavin T.

Phospholipid membranes, like other negatively charged molecules, have been shown to facilitate tau fibril formation *in vitro*. In light of perturbations to K18 membrane binding by all three PTM-mimetic mutations, we examined their effects on membrane-enhanced filament formation using the thioflavin T (ThT) aggregation assay. Incubation of 200 μM unmodified K18 in the presence of 4 mM phosphatidylserine isolated from porcine brain (BPS) lipid vesicles while shaking at 1000 RPM at 37 °C results in a steady increase in ThT fluorescence, which plateaus after about 24 h, with a T50 value (time to half-maximal fluorescence) of 8.72 h (Fig. 10*A*). In contrast, under identical conditions in the absence of vesicles, ThT fluorescence increases more slowly, with a T50 of 12.77 h (Fig. S12). For the three PTM mimetics (Fig. 10*B*), we observe an increased T50 (Table 2), indicating less enhancement of membrane-induced fibril. While these data are consistent with the decrease in membrane binding observed for all three mutants, the magnitude of the effect on T50 does not correlate with the decrease in membrane binding. Negative-stain images of the reactions confirm the presence of fibrils for all variants and do not reveal any obvious difference in fibril morphology (Fig. S11).

**Table 2:** PTM mimetics aggregated in the presence of 4 mM BPS vesicles

Variable/Variant	K18	K280Q	Y310E	K311E
*T*_50_ (h)	7.55	20.71	26.80	11.98
*T*_lag_ (h)	6	24	24	12

*T*_50_, determined as the half-time to max fluorescence of 200 μM K18 and *T*_lag_, determined as the first time point at which fluorescence exceeds 10% of the endpoint fluorescence.

## Discussion

The tau MBD is involved in interactions with a variety of binding partners, including itself, tubulin, MTs, and membranes, with both functional and pathological roles. While many tau PTMs are found outside the MBD, including the most common tau modification, Ser/Thr phosphorylation, PTMs within the MBD may be expected to have strong effects on tau interactions, including self-assembly. This view is supported by recent structures of tau fibrils derived from human brains (62, 63, 82, 83), in which the intimate arrangements of beta-strand within the MBD that determine the specific fold of fibrils associated with different disease could clearly be perturbed by PTMs. Indeed, it has been proposed that the presence of PTMs may drive the formation of different fibril structures by favoring certain conformations over others (84).

We recently reported the discovery of a novel tau PTM located within the MBD, succinylation of lysine 311, which was detected in nearly all AD brains studied and never in disease-free control brains (13). While the etiological role, if any, of this modification remains unclear, its location within the MBD, and within the most potent known PHF nucleating motif, the PHF6 hexapeptide, suggests that this PTM may influence tau interactions in important ways. Here, we set out to examine the effects of K311 succinylation on tau interactions with tubulin and membranes and on membrane-induced tau oligomer and fibril formation. Two other PTMs are also known to occur with the two MBD hexapeptide motifs, acetylation of lysine 280 within the PHF6^∗^ motif and phosphorylation of tyrosine 310 within the PHF6 motif. We included these PTMs in our study both for the purpose of comparison with K311 succinylation and because they have been previously characterized to some extent.

While phosphorylation is strictly mediated by enzymatic activity in eukaryotes, lysine acylation can occur nonenzymatically *via* reactions involving reactive metabolic acyl-CoA intermediates (85). We examined the nonenzymatic acetylation and succinylation of the tau MBD fragment K18 upon incubation with acetyl-CoA or succinyl-CoA using NMR, capitalizing on the shift in the lysine epsilon amino group upon modification. Succinylation was efficient, resulting in modification of multiple lysine residue equivalents, whereas acetylation was much less efficient, with less than one lysine equivalent modified under our conditions. To monitor MBD–tubulin interactions, we mixed K18 with stoichiometric amounts of T2R, a dimer of tubulin heterodimers stabilized by a stathmin-like domain to prevent complications from tubulin polymerization or depolymerization (68). T2R has previously been used to examine tau–tubulin interactions (44), and the stathmin-like domain binds in a manner that does not occlude the tau-binding sites revealed in recent cryo-EM structures of MT-bound tau (43). Our NMR data reveal T2R interactions throughout the K18 sequence, consistent with previous reports (44), and enable us to define two regions, termed R2_T2R_ and R3_T2R_, that show the strongest binding.

Unsurprisingly, succinylation at multiple lysine residues lead to a dramatic decrease in K18–T2R interactions (Fig. 3*A*). Acetylation of K18 also lead to a measurable decrease in T2R binding (Fig. 3*B*), despite some uncertainty regarding the extent of modification. In both cases, all regions of K18 were affected, consistent with modification at multiple different sites. In contrast to nonenzymatic modification, phosphorylation of K18 by the c-Abl results in quantitative modification of Y310 (33), the only tyrosine residue in the tau MBD. Y310 phosphorylation also resulted in reduced binding of K18 to MBD, but the effect was more pronounced for the R3 repeat and most pronounced for the R3_T2R_ motif (Fig. 3*C*).

Because site-specific succinylation of proteins or long polypeptides is not easily achievable, especially at quantities required for NMR, we chose to investigate the site-specific effects of K311 succinylation and K280 acetylation using shorter peptides, where these PTMs can be synthetically incorporated. We used two peptides comprising tau residues 265 to 290 and 296 to 321, which include the R2_T2R_ and R3_T2R_ motifs, respectively, because these were previously used in binding studies with tubulin (13, 41, 42). We included a Y310 phosphorylated peptide for completeness and for comparison with the results obtained using Y310 phosphorylated K18. STD NMR, which is effective at monitoring the interactions of small ligands with large receptors, confirmed binding of both peptides to T2R, as expected. All three PTMs resulted in decreased signal in STD spectra, indicating weaker binding, and our attempts to quantify these effects (Fig. 4) suggest that Y310 phosphorylation severely reduced peptide binding to T2R, to the point where the data could not be meaningfully fit using the experimentally accessible concentrations. Succinylation of K311 and K280 acetylation also resulted in a trend toward reduced binding, though to a lesser extent.

Our results suggest that the R3_T2R_ motif (296–321) and R2_T2R_ motif peptide (265–290) bind with similar affinities, consistent with early studies of different contributions of the four tau repeats to MT binding (73, 86). Our data also reproduce previous observations that show signals from protons in the Y310 side chain are particularly strong in STD spectra of tubulin-bound tau peptides (41), suggesting close contacts between this side chain and tubulin. This likely explains both the strong perturbing effect of the Y310E mutation, which presumably removes these contacts, and the weaker effect of the K311E mutation, which may perturb interactions associated with the lysine side chain, but leaves the strong tyrosine side-chain interactions intact. The R2_T2R_ motif does not contain an equivalent tyrosine residue to Y310, but unlike the R3_T2R_ motif, it contains an additional lysine at position 281, which has been reported to contribute strongly to tau–tubulin interactions (87). Modification of the K280 lysine side chain leads to a disruption of tubulin binding similar to that observed upon succinylation of K311 in the R3_T2R_ motif, possibly because K312 continues to support local binding. Notably, Y310 phosphorylation in the context of K18 also leads to a disruption of local binding of the R3_T2R_ motif, but because K18 includes the R2_T2R_ motif as well as other sites of T2R interaction, the global effect on binding is much less in the context of K18 than for the R3_T2R_ motif peptide alone. Our observations are also consistent with a previous report indicating that Y310 phosphorylation contributes to reduced binding of c-Abl phosphorylated full-length tau (33).

The effects of protein PTMs are often explored using PTM-mimetic point mutations, especially for work performed *in situ* or *in vivo*, where site-specific PTMs may be impossible or extremely challenging to generate. Ideally, use of such mimetics should be evaluated and validated by experiments comparing their effects with those of authentic PTMs, as in some cases, the mimetics do not reproduce the effects of the authentic PTMs (88, 89). To facilitate future studies of the effects of K311 succinylation *in situ* and *in vivo*, we compared the effects of the succinylation mimetic K311E mutation on the binding of K18 to T2R. Because the K280Q mutation has been previously used to mimic K280 acetylation in various models (24, 77), but its effects on tubulin binding have not been carefully evaluated, we included studies of this mutant, as well as of the phosphomimetic Y310E mutation. We find that each of the three PTM-mimetic mutations results in a local decrease in binding, as shown by increased average NMR intensity ratios for the R2_T2R_ (K280Q) and R3_T2R_ (Y310E and K311E) motifs, although the effect for K280Q did not reach statistical significance (Fig. 5). These observations are in agreement with our STD data for the synthetic peptides, where each PTM was also observed to perturb local binding, suggesting some fidelity between the mimetics and the actual PTMs. Also consistent with the STD data, the effects of the Y310E mutation were stronger than those of the K311E mutation. Notably, the effects of the Y310E mutant were considerably smaller than those of Y310 phosphorylation (Fig. 3*C* *versus* Fig. 3*F*), indicating that the phosphomimetic mutant does not fully capture the effects of the authentic PTM.

Our data indicate that when considering tau–tubulin interactions, PTM-mimetic mutations, K280Q, Y310E, and K311E, produce effects that are similar in nature to, but possibly weaker in extent than those of the authentic PTMs at these sites. This may result from differences in the detailed properties of the mutant side chains, glutamine, glutamate, and glutamate compared with the PTM-modified side chains, acetyl-lysine, succinyl-lysine, and phosphotyrosine. The mutants may mimic changes in electrostatic charge but not the detailed steric features of the PTM. Notably, our results are also generally consistent with previous reports that both K280Q and K311D mutations reduce K18-mediated tubulin assembly *in vitro* (24, 87). We next considered the potential effects of tau on interactions with other cellular components, namely membranes, because such interactions may contribute not only to tau function (57, 90) but also are thought to potentially nucleate tau self-assembly *in vivo* (53, 78, 79, 80, 81). While tau–tubulin interactions, like other protein–protein interactions, may be highly specific and sensitive to the specific steric packing details, tau–membrane interactions are likely to be mediated by more general hydrophobic and electrostatic interactions and less sensitive to such details. In such cases, PTM-mimetic mutations are more likely to capture the effects of actual PTMs (39).

We therefore set out to examine the effects of each PTM-mimetic mutation on the binding of K18 to SUVs composed of 4:1 POPC:POPS, since tau interacts with such vesicles without triggering rapid aggregation. We first mapped the membrane-interacting regions of unmodified K18 and discovered that in addition to the previous membrane-binding amphipathic helices within each tau repeat, the N-terminal regions of R2 and R3, including each hexapeptide motif as well as three subsequent residues (referred to as PHF6^∗^+ and PHF6+), also exhibit tight binding to SUVs. Although our studies of tau–micelle interactions described restricted mobility in these regions upon micelle binding (58), and previous work reported membrane binding by isolated PHF6 or R3 peptides (33, 91), membrane binding by these regions within intact MBD constructs has not previously been reported. We therefore validated binding of the PHF6 region using Trp mutagenesis and fluorescence spectroscopy (Fig. 7*B*), after first verifying the ability of this method to detect binding and helical structure in the previously characterized helix 3 region (Fig. 7*A*).

Having mapped and confirmed regions that contribute to K18 membrane binding, we examined the effects of PTM-mimetic mutations. The K280Q mutant reduced binding to membranes for the helix 1 and helix 2 regions as well as the PHF6^∗^+ region, which contains the mutation site and is situated between the two helical regions. This indicates a decrease in local binding around the site of the mutation, consistent with replacing a positively charged lysine, which would be expected to interact favorably with the negatively charged membrane surface, with a neutral glutamine side chain. In contrast, both the Y310E and K311E mutations resulted in more severe perturbations to K18 membrane binding, that included all four helices and both the PHF6^∗^+ and PHF6+ regions, suggesting a global decrease in the affinity of the entire polypeptide for membranes. These stronger effects are likely due in part to the introduction of a negative charge into a membrane-interacting region, leading to electrostatic repulsion between the glutamate side chain and the negatively charged membrane surface. At the same time, the propagation of this effect through the K18 sequence suggests that the interactions of the PHF6+ region with membranes is an important contributor to the overall membrane affinity of the MBD. This conclusion is supported by observations that Y310 phosphorylation strongly reduced membrane binding of K18 (33).

Incubation of full-length tau or tau K18 with negatively charged lipid vesicles leads to the formation of membrane-induced oligomers that are toxic to cultured neurons (53). Therefore, understanding how PTMs alter membrane-induced oligomer formation is of considerable interest. We find that all three PTM mimetics conserve the ability of K18 to form membrane-induced oligomers. However, the elution profiles of the mutant oligomers differ from those formed by the unmodified protein, with the mutant oligomers eluting later. Notably, Y310 phosphorylation also leads to a similarly increased elution time for membrane-induced K18 oligomers (33), indicating that in this case, the Y310E phosphomimetic successfully mimics the authentic PTM. Changes in elution time could be caused by differences in either the MW of the oligomers or of their shape. To assess whether the composition of the structured core of the oligomers, which for unmodified K18 consists of the PHF6^∗^+9 and PHF6+9 regions, was altered by the mimetic mutations, we examined solution-state NMR spectra of oligomer preparations, in which regions immobilized in the oligomer core exhibit decreased signal intensities resulting in lower intensity ratios. The intensity ratio profiles of the mutants were unaltered compared with those of the unmodified protein (Fig. 9, *B* and *C*), indicating that the composition of the oligomer core is preserved in the mutants. This suggests that either the MW of the oligomers or the arrangement of the molecules within the core is responsible for the shift in the elution volume.

In addition to differences in their elution volumes, the elution profiles of the three PTM mimetics consistently included a void peak that was more pronounced than that observed for the unmodified protein. This peak consists primarily of intact lipid vesicles that are not disrupted by oligomer formation (53) and is therefore consistent with the lower affinity of the mutants for vesicles. Indeed, the oligomer peaks for the Y310E and K311E mutants, which exhibited the greatest decrease in membrane biding, were consistently smaller than those for the WT protein, indicating less efficient membrane-induced oligomer formation for these mutants.

Finally, we examined the effects of the PTM mimetics on membrane-facilitated assembly of K18 into fibrils. As measured by ThT fluorescence, all three mutations lead to slower formation of fibrils upon incubation with lipid vesicles while shaking. Because membrane-facilitated aggregation presumably requires membrane interactions, this observation is consistent with the reduced affinity of the mutants for lipid vesicles. The magnitude of the effect, however, did not correlate with reductions in membrane binding, as the K311E mutant, which perturbed membrane binding to a much greater extent than the K280Q mutant, had a similar or lesser effect on membrane-induced fibril formation. This suggests that these mutations may exert effects on additional steps beyond membrane binding, as might be expected once the specific interactions observed in fibril structures begin to form. Comparing membrane-facilitated aggregation with heparin-facilitated aggregation, it is also clear that these processes differ somewhat, as the latter is enhanced by the K280Q mutation (87), though it is delayed by the K311D mutation (65, 92) and by Y310 phosphorylation (33).

## Conclusions

We have characterized the effects of a novel disease-associated tau PTM, K311 succinylation, and shown that it locally perturbs the binding of the tau MBD to tubulin, to an extent similar to that of K280 acetylation but to a lesser extent than Y310 phosphorylation. We have also shown that point mutations designed to mimic these three PTMs exert effects on tubulin binding that are similar to those exerted by the authentic PTMs, but at least for Y310E, smaller than the effects of the authentic PTM. We have demonstrated that all three PTM mimetics decrease membrane binding by the tau MBD, with a more pronounced effect for Y310E and K311E, and also alter the hydrodynamic properties of membrane-induced tau oligomers by changing either their size or their conformation. Decreased membrane binding by the mutants is also associated with less efficient formation of oligomers and slower formation of membrane-facilitated fibrils. Our results have important implications for future studies of tau succinylation, establishing the K311E mutant as a tool that likely captures the effects of this PTM on several tau interactions. They also expand our understanding of the effects of K280 acetylation and Y310 phosphorylation on tau interactions. Further studies will be required to determine the effects of these and other tau PTMs on the detailed structures of tau fibrils formed under different conditions, as well as on other important processes related to tau disease propagation, such as interactions with tau cell surface receptors (93, 94).

## Experimental procedures

### Recombinant protein expression and purification

Recombinant protein was expressed in *Escherichia coli* BL21/DE3 cells (Novagen) transfected with plasmids for the tau fragment K18 under the control of a T7 promoter as previously described (64). For PTM-mimetic mutants, QuikChange Site-Directed Mutagenesis (Agilent Technologies) was used to introduce the mutations into K18. Overexpression was induced with 0.5 mM IPTG at an absorbance at 600 nm during 3 h at 37 °C. Cells were lysed by sonication in 3 mM urea,1 mM EDTA, 1 mM DTT, 10 mM Tris, and 1 mM PMSF followed by ultracentrifugation at 40,000 rpm in a Beckman ultracentrifuge using a Ti 50.2 rotor. The supernatant was dialyzed against 25 mM Tris, 20 mM NaCl, 1 mM EDTA, and 1 mM DTT before being purified by cation exchange chromatography and eluted by an NaCl gradient. Fractions containing tau K18 were pooled and dialyzed against 5% acetic acid before being further purified by reverse-phase high-performance liquid chromatography on a C4 column and eluted by an acetonitrile gradient with 1% trifluoroacetic acid. Purified protein was dialyzed against distilled water before being lyophilized and stored at −20 °C. Purity was confirmed by SDS-PAGE.

### RB3-SLD expression and purification

Stathmin-like RB3 domain (RB3_SLD_) was expressed in *E. coli* BL21/DE3 cells (Novagen) transfected with PET-3d. Overexpression was induced with 0.5 mM IPTG, and growths were monitored at an absorbance at 600 nm during 3 h at 37 °C. Cells were lysed by sonication in 20 mM Tris–HCl, 1 mM EGTA, and 1 mM DTT at pH 8.0, followed by ultracentrifugation at 20,000*g* in a Beckman ultracentrifuge using a Ti 50.2 rotor. The supernatant underwent thermal denaturation (80 °C for 15 min and then 10 min on ice) and was recentrifuged, followed by nucleic acid precipitation with 20 mM spermine hydrochloride at pH 7.0 for 30 min at 4 °C with gentle agitation, and centrifuged once more (1 h at 100,000*g*). The supernatant was dialyzed against 20 mM Tris–HCl, 1 mM EGTA at pH 8.0 before further purification by anion exchange, and eluted by an NaCl gradient. Purity was confirmed by SDS-PAGE before concentrating the purified protein and buffer exchanging into 50 mM phosphate and 0.1 M NaCl at pH 7.0 with a gel filtration column. Final protein product was flash frozen and stored at −80 °C until use. SDS-PAGE analysis was used to estimate the concentration of final protein.

### Tubulin and T2R preparation

Tubulin protein isolated from porcine brain (97 or >99% pure) was purchased from Cytoskeleton, Inc, as a lyophilized powder. Stathmin-like RB3 domain (RB3_SLD_) binds to heterodimeric tubulin with a 1:2 stoichiometry, forming a longitudinal dimer of tubulin dimers. For ^1^H,^15^N HSQC NMR experiments, lyophilized tubulin was dissolved into a solution of 48 μM ^15^N-labeled K18 (unmodified, modified, or mutant) containing 53 μM RB3 for a final T2R concentration of 50 μM in a buffer containing 25 mM Tris-d11, 25 mM NaCl, 2.5 mM EDTA, 1.5 mM DTT, and 10% heavy water (deuterium oxide [D_2_O]) at pH 6.7. For ^1^H STD NMR experiments, 10 μM T2R was used for samples containing 0.044 to 2.8 mM peptide. Peptide was directly dissolved into buffer or water and added to 10 μM T2R in a buffer containing 25 mM Tris-d11, 25 mM NaCl, 2.5 mM EDTA, 1.5 mM DTT, and 10% D_2_O at pH 6.7.

### Nonenzymatic succinylation of tau K18

Lyophilized ^15^N-labeled K18 was dissolved into 80 mM PIPES, 2 mM MgCl_2_, 1 mM GTP, and 0.5 mM EGTA, pH 6.8 to a final protein concentration of 100 μM. The samples were centrifuged in a spin filter to remove large aggregates. The filtered protein was mixed with succinyl-CoA (final concentration of 2.8 mM) and incubated overnight at 37 °C. The reaction was monitored with ^15^N–^1^H HSQC NMR *via* the succinyl-lysine peak at 125.72 ppm ^5^N and 7.94 ppm ^1^H until completion. The samples were concentrated by ultracentrifugal filters to remove succinyl-CoA, buffer exchanged into the required buffer, and immediately used for binding assays.

### Nonenzymatic acetylation of tau K18

Lyophilized ^15^N-labeled K18 was dissolved into 80 mM PIPES, 2 mM MgCl_2_, 1 mM GTP, and 0.5 mM EGTA, pH 6.8 to a final concentration of 100 μM. The samples were centrifuged in a spin filter to remove large aggregates. The filtered protein was mixed with acetyl-CoA (a final concentration of 2.8 mM) and incubated overnight at 37 °C. The reaction was monitored with ^15^N–^1^H HSQC NMR *via* the acetyl-lysine peak at 127.45 ppm ^15^N and 8.01 ppm ^1^H until completion. The samples were concentrated by ultracentrifugal filters to remove acetyl-CoA, buffer exchanged into the required buffer, and immediately used for binding assays.

### Quantification of lysine acetylation and succinylation

Acetylated lysine ϵ-amino resonances appear at ∼127.5 ppm in the ^15^N dimension and ∼8.05 ppm in the ^1^H dimension, whereas succinylated lysine ϵ-amino resonances appear at ∼125.5 ppm in the ^15^N dimension and ∼7.95 ppm in the ^1^H dimension. For each acetylation or succinylation reaction, the peak volume for the modified lysine ϵ-amino group was normalized by that of the unmodified K311 backbone NH peak to estimate the number of modified lysines. This was then divided by the total number of lysine residues in K18 construct to determine the fraction of modified lysine residues.

### C-Abl tyrosine phosphorylation

Phosphorylation of purified ^15^N-labeled K18 was accomplished using recombinant c-Abl kinase (kindly gifted to us by Markus Seeliger, Stonybrook University). Purified K18 was incubated with c-Abl kinase at a 1:40 kinase:protein (w/w) ratio in 50 mm Tris, 5 mm MgCl_2_, 1 mm DTT, 1.5 mM PMSF, 20 mm Na_3_VO_4_ (phosphatase inhibitor), 5 mm ATP, and pH 7.2. Reactions were performed for 24 h at 30 °C without agitation. The reactions were followed using NMR (33).

### NMR spectroscopy

All NMR spectra were collected on a Bruker AVANCE 600-MHz spectrometer equipped with a cryogenic triple resonance probe. ^1^H,^15^N HSQC NMR was collected at 20 °C with 1024 complex points in the ^1^H dimension, 256 complex points in the ^15^N dimension, a spectral width of 26 ppm, and an offset of 4.7 ppm in the ^1^H dimension. Assignments were based on our previously published assignments for K18 (58). Intensity values were extracted for resonances of each residue in concentration-matched free-state (I_0_) and bound-state (I) samples. Protein binding was assessed *via* intensity ratios (I/I_0_) for tau–T2R interactions and tau–lipid interactions. Intensity ratios for oligomeric preparations of K18 were calculated as the ratio of intensities in the oligomeric samples (I) to monomeric samples (I_0_). Samples for ^1^H,^15^N HSQC NMR experiments with T2R were prepared by resuspending lyophilized protein in Tris-d_11_ buffer composed of 25 mM Tris-d_11_, 25 mM NaCl, 2.5 mM EDTA, and 1.5 mM DTT with 10% D_2_O at pH 6.7. Samples for ^1^H,^15^N HSQC NMR experiments with vesicle binding and of oligomeric complexes were performed in 10 mM Hepes, 100 mM NaCl, 2.5 mM DTT, 10% D_2_O, and pH 7.4.

### STD NMR

STD spectra were recorded at 10 °C on a 600 MHz spectrometer equipped with a cryoprobe by using a series of 40 equally spaced 50 ms Gaussian-shaped pulses, 4096 scans, a saturation time of 2 s, and on/off-resonance frequencies set to −0.5 and 60 ppm, respectively. Samples for ^1^H STD NMR were dissolved into GPEM buffer (80 mM PIPES, 2 mM MgCl_2_, and 0.5 mM EGTA, at pH 6.9). Spectra were processed and analyzed using Topspin 3.1 (Bruker). We quantify the binding of tau peptides to T2R with a series of STD titration experiments (95). STD NMR spectra of the ligand with and without T2R were recorded. The concentration of ligand (tau peptide) ranged from 0.04 to 2.8 mM; T2R concentration was maintained at 10 μM. From STD NMR spectra, STD signals were integrated and summed for both STD_diff_ and STD_ref_ over three regions, 8.565 to 7.771, 7.584 to 7.384, and 7.073 to 6.681 ppm. From STD_diff_ and STD_ref_, STD AFs (STD_AF_) were calculated according to Equation 1, where [L] is the concentration of peptide (0.04–2.8 mM) and [R] is the concentration of T2R (10 μM). Binding affinities were determined using STD experiments at increasing peptide concentrations (0.04–2.8 mM) for constant T2R concentrations of 10 μM. STD-AF values (at a fixed saturation time) of a peptide were fitted according to Equation 2, where αSTD represents the maximum STD AF (72). For peptide concentration of 0.7 μM and T2R concentration of 10 μM, we generated STD build-up curves by recording the STD difference signal at increasing saturation times of 0.1 to 4 s. We fit Equation 3 to these data to determine the initial slope (STD_0_), from which we determined an appropriate saturation time for our experiments to minimize any artifacts from the bound state (95, 96) and the influence of any rebinding effect (95, 96).

(1) $${\text{STD}}_{\text{AF}}=\frac{{\text{STD}}_{\text{diff}}}{{\text{STD}}_{\text{ref}}}*\;\frac{\left[\text{L}\right]}{\left[\text{R}\right]}$$

(2) $${\text{STD}}_{\text{AF}}\left(\left[\text{L}\right]\right)=\;\frac{\text{αSTD}*\left[\text{L}\right]}{K_{d}+\left[\text{L}\right]}$$

(3) $${\text{STD}}_{\text{AF}}\left(t\right)=\;{\text{STD}}_{\text{max}}\left(1-\;{\text{e}}^{\left(-K_{\text{sat}}*t\right)}\right)$$

$${\text{STD}}_{0}={\text{STD}}_{\text{max}}*\;K_{\text{sat}}$$

### Synthetic peptides

Synthetic peptides were purchased from Biomatik. Peptides used in these studies include:

296 to 321 (NIKHVPGGGSVQIVYKPVDLSKVTSK)

265 to 290 (NLKHQPGGGKVQIINKKLDLSNVQSK)

Acetyl-K280 265 to 290 (NLKHQPGGGKVQIIN {acetyl-K} KLDLSNVQSK)

Succinyl-K311 296 to 321 (NIKHVPGGGSVQIVY {succinyl-K} PVDLSKVTSK)

Phos-Y310 296 to 321 (NIKHVPGGGSVQIV {phospho-Y} KPVDLSKVTSK)

### ThT fluorescence measurements

Aggregation was induced by incubating BPS vesicles with K18 or the mimetic constructs at a molar ratio of 1:20 (protein:phospholipid) in 10 mM Hepes, pH 7.4, 100 mM NaCl, and 2.5 mM DTT at 37 °C. ThT was added immediately prior to shaking at 1000 rpm. The data were collected in triplicates at time intervals (0, 6, 12, 24, 30, 36, 48, and 72 h) using a microplate fluorescence reader (Molecular Devices). The excitation and emission wavelengths were 440 and 490 nm, respectively.

Half time to maximal fluorescence (*T*_50_) of kinetic aggregation data was used as a measure of seeding competence for each tau construct. *T*_50_ was determined by fitting aggregation data to Equation 3 (86), where *y* is the fluorescence intensity; A2 and A1 are the initial and maximum intensity signals, respectively; *T* is the measurement time, and d*T* is the time constant. Data were graphed and analyzed using GraphPad Prism version 8.0 for Mac OS X, GraphPad Software, www.graphpad.com.

(4) $$y=A2+\;\frac{A1-A2}{1+\;{\text{e}}^{\left(T-T_{50}\right)/\text{d}T}}$$

### Preparation of tau–phospholipid oligomer complexes

The protein–phospholipid complexes were prepared by incubating BPS vesicles with K18 or the mimetic constructs at a molar ratio of 1:20 (protein:phospholipid) in 10 mM Hepes, pH 7.4, 100 mM NaCl, and 2.5 mM DTT for at least 24 h at 37 °C. The resulting protein–phospholipid complexes were separated from remaining vesicles and soluble protein by size-exclusion chromatography using a Superose 6 column (GE Healthcare).

### Vesicle preparation

BPS, phosphatidylserine, and phosphatidylcholine were purchased from Avanti Polar Lipids, Inc. For preparation of large unilamellar vesicles, phospholipids or phospholipid mixtures in chloroform were dried using a nitrogen stream to form a thin film on the wall of a glass vial. Any remaining chloroform was removed by placing the vial under vacuum. The phospholipids were resolubilized in 10 mM Hepes, pH 7.4, 100 mM NaCl, and 2.5 mM DTT to the desired final concentration. The solution was then passed through an Avestin LiposoFast extruder (Avestin, Inc) with a membrane of pore size 0.2 μM. Size and homogeneity of the resulting vesicles were assessed by dynamic light scattering. For the preparation of SUVs, phospholipids were not passed through an extruder, instead lipids were sonicated until clear. The clarified lipids were ultracentrifuged at 60,000 rpm (250,000*g*) for at least 1 h at 4 °C. All phospholipids were used immediately following preparation for assays.

### Measuring lipid binding with fluorescence

For acrylodan fluorescence, we used site-directed mutagenesis to introduce cysteines at positions of interest for probing membrane interactions. Protein was resuspended in purification buffer (25 mM Tris, 100 mM NaCl, 1 mM EDTA, 1 mM DTT, and pH 7.4) at a concentration of 600 μM and a volume of 1.3 ml and left at room temperature for 15 to 30 min. The sample was filtered through a 100 kDa centrifugal spin column at 4000 rpm on a benchtop centrifuge at 4 °C for 15 min or until the sample completely ran through. The protein sample was buffer exchanged into labeling buffer (20 mM Tris, 50 mM NaCl, pH 7.4) with a PD-10 column. About 80 μl of 50 mM acrylodan was added to the buffer-exchanged protein sample (10 μl at a time). The sample was covered in foil and placed on a rotator for 4 h at room temperature. Residual acrylodan was removed by spinning down the sample for 5 min at 14,000 rpm (18,000*g*) on a Beckman Microfuge 18 centrifuge. Labeled protein was buffer exchanged into phosphate buffer (20 mM Na_2_HPO_4_, 20 mM KCl, 1 mM MgCl_2_, 0.5 mM EDTA, and pH 7.2) with a PD-10 column. About ∼1:1500 (protein:lipid) molar ratio samples were made; we worked with 20 μM of tau protein with 30 mM SUV lipids. Phosphate buffer was used as a blank for free-state protein samples. A lipid sample containing 30 mM liposome in buffer without protein was used as a blank for bound-state protein samples to subtract any noise signal emerging from lipid or buffer, leaving only the protein fluorescence signal. Plate reader parameters were set to excitation wavelength of 390 nm, cutoff of 420 nm, and emission of 400 to 600 nm. Bound-state and free-state proteins were scanned alongside the controls.

For tryptophan fluorescence, site-directed mutagenesis was used to introduce tryptophan at specific sites of interest. Tryptophan emission was probed upon excitation at 295 nm. For our experiments, we used 20 μM tau protein and 30 mM POPC:POPS SUV lipids. Buffer was used as a blank for free-state protein samples. A lipid sample containing 30 mM POPC:POPS in buffer was used as a blank for bound-state protein samples. Plate reader parameters were set to excitation wavelength of 295 nm, cutoff of none, and emission of 300 to 500 nm. Bound-state and free-state protein samples were scanned.

Fluorescence emission spectra from 300 to 450 nm (RFU *versus* λ [nm]) should show a peak around 355 nm for Trp-labeled free-state samples and around 520 nm for acrylodan-labeled free-state samples. Samples in the presence of lipid vesicles may show a shift in the peak maximum toward lower wavelengths, reflecting insertion of the fluorophore into the membrane. The differences in delta lambda max for free state *versus* bound state were calculated. Delta lambda max results from a change in the environment of the fluorophore. Small values of delta lambda max indicate small differences in fluorophore environment in the presence of lipids, suggesting no membrane interaction or insertion at this position. Larger values suggest membrane interaction or insertion. For multiple successive residues, a plot of delta lambda max *versus* residue position was constructed. A periodic function (Equation 5) was fit to the data to estimate the periodicity across a given region. Any periodicity observed in such a plot may reflect a periodicity in the environment of successive residues in the targeted protein segment, which may reflect, for example, the underlying periodicity of secondary structure elements. A periodicity of 3.6 would be consistent with an α-helix secondary structure.

(5) $$\left(\Delta {\text{λ}}_{\text{max}}(\text{n})=\text{a}+\text{b∗cos}(2\text{πn}/\text{N}+\text{c}\right)$$

### Negative-stain electron microscopy

A Pelco Easi-glo discharge unit was used to glow discharge a carbon-coated mesh copper grid. Afterward, 5 μl of the sample (fibril or oligomer) was incubated on the grid for 1 min. The grid was washed with water three times, washed with stain (uranyl acetate 1% w/v) twice, incubated with stain for 30 s, washed with water, and blotted with filter paper to remove excess liquid from the grid. The grid was allowed to dry before proceeding with EM imaging using a JEOL JEM 1400 transmission electron microscope operated at 100 kV and equipped with an Olympus-SIS Veleta side-mount 2K × 2K digital camera.

### Statistics

One-way ANOVA followed by a Dunnett's multiple comparison test was performed for the comparison of multiple groups (PTM mimetics) to a single control group (K18). Statistical significance was reported at *p* < 0.05. (*p* < 0.033 [∗], *p* < 0.002 [∗∗], and *p* < 0.001 [∗∗∗]). Data were graphed and analyzed using GraphPad Prism version 8.0.

## Data availability

All data can be shared upon request.

## Supporting information

This article contains supporting information (58, 81, 97).

## Conflict of interest

The authors declare that they have no conflicts of interest with the contents of this article.
