# Supplementary material for: Post-translational modifications within tau paired helical filament nucleating motifs perturb microtubule interactions and oligomer formation
Source: J Biol Chem. 2021 Nov 24;298(1):101442. doi: 10.1016/j.jbc.2021.101442 (PMC8741514; doi:10.1016/j.jbc.2021.101442)
Supplement: Supplemental Figures S1–S12 [file mmc1.docx]

**Title**

Post-translational modification within PHF nucleating motifs of tau modulate interactions and aggregation

**Authors**

Diana M. Acosta

Feil Family Brain and Mind Research Institute

Department of Biochemistry and Program in Structural Biology

Weill Cornell Medicine, New York, NY 10065

dia2018@med.cornell.edu

Chiara Mancinelli

Department of Biochemistry and Program in Structural Biology

Weill Cornell Medicine, New York, NY 10065

cdm4001@med.cornell.edu

Dr. Clay Bracken

Department of Biochemistry

Weill Cornell Medicine, New York, NY, USA

wcb2001@med.cornell.edu

Dr. David Eliezer **– *Corresponding Author**

Feil Family Brain and Mind Research Institute

Department of Biochemistry and Program in Structural Biology

Weill Cornell Medicine, New York, NY 10065

dae2005@med.cornell.edu

**List of material included:**

- Figure S1: Monitoring succinylation and acetylation modification at the lysine epsilon amino site by NMR HSQC spectrum.
- Figure S2: Post-translational modifications of ^15^N-K18 alter binding to T2R.
- Figure S3: Monitoring tau-T2R interactions with saturation transfer difference NMR
- Figure S4: ^1^H,^15^N HSQC of tau-T2R interactions.
- Figure S5: Intensity ratio plots of tau-T2R interactions of post-translational modification mimetics.
- Figure S6: Comparison of Mimetics to PTM’s on tau-T2R binding.
- Figure S7: Acrylodan fluorescence of tau labeled at residues 315-322.
- Figure S8: Lipid Binding Curves of Helix 1-4 for K18 and PTM mimetic K18.
- Figure S9: Negative stain electron microscopy (EM) of tau oligomers.
- Figure S10: Intensity ratio plots of tau-BPS phospholipid complexes.
- Figure S11: Negative Stain EM images of tau Fibrils.
- Figure S12: Self-aggregation of tau.

**Figure S1: Monitoring succinylation and acetylation at the lysine epsilon amino site by NMR HSQC spectra.** (A) Succinylation reactions of tau K18 (99 uM) with succinyl-CoA (2.8 mM) results in a modified epsilon amino resonance peak at position ~125.5 ppm in the ^15^N dimension and ~7.95 ppm in the ^1^H dimension. (B) Acetylation reactions of tau K18 (99 uM) with acetyl-CoA (2.8 mM) results in a modified epsilon amino resonance peak at position ~127.5 ppm in the ^15^N dimension and ~8.05 ppm in the ^1^H dimension.

**Figure S2: Post-translational modifications of ^15^N-K18 alter binding to T2R.** ^1^H,^15^N HSQC spectra were recorded for tau K18 constructs (42 uM) of (A) unmodified, (B) succinylated, (C) acetylated and (D) phosphorylated in the absence and presence of T2R (50 uM). Intensity ratios were calculated for assigned resolved resonances, based on previous assignments (58, 97), as the intensity in the presence of T2R normalized by the corresponding intensity in the absence of T2R. MBD repeats are indicated in alternating grey shading.


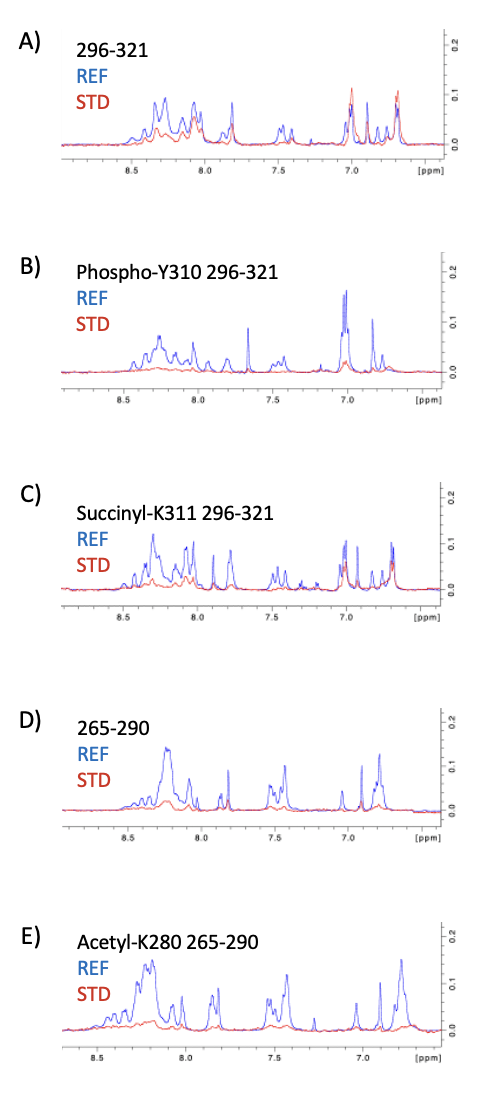


**Figure S3: Monitoring tau-T2R interactions with saturation transfer difference NMR**. Tau peptides (2.8 mM) encompassing the PHF6 region (296-321) (A), or the PHF6* region (265-290) (D) bind to T2R (10 uM) as evident by present STD signals (red). Decreases in binding are observed when single sites are modified by (B) phosphorylation at residue Y310, (C) succinylation at residue K311, and (E) acetylation at residue K280 as evident by their decreased STD signals (red) compared to their unmodified counterpart.

**Figure S4: ^1^H,^15^N HSQC of tau-T2R interactions.** ^1^H,^15^N HSQC spectra were recorded for (A) unmodified tau K18, (B-D) post-translationally modified K18 and (E-G) post-translational modification mimetics. ^1^H,^15^N HSQC spectra were collected in the absence (black resonances) and presence of T2R complex (50 uM) (red resonances). Assigned resonances were used for quantification.

**Figure S5: Intensity ratio plots of tau-T2R interactions of post-translational modification mimetics.** (A) Succinylation mimetic K311E, (B) Acetylation mimetic K280Q, and (C) Phosphorylation mimetic Y310E. Resolved resonances from ^1^H,^15^N HSQC spectra (Figure S4) in the absence and presence of T2R (50 uM) were used to calculate intensity ratios, based on previous assignments (58,97), as the intensity in the presence of T2R normalized by the corresponding intensity in the absence of T2R. MBD repeats are indicated in alternating grey shading.


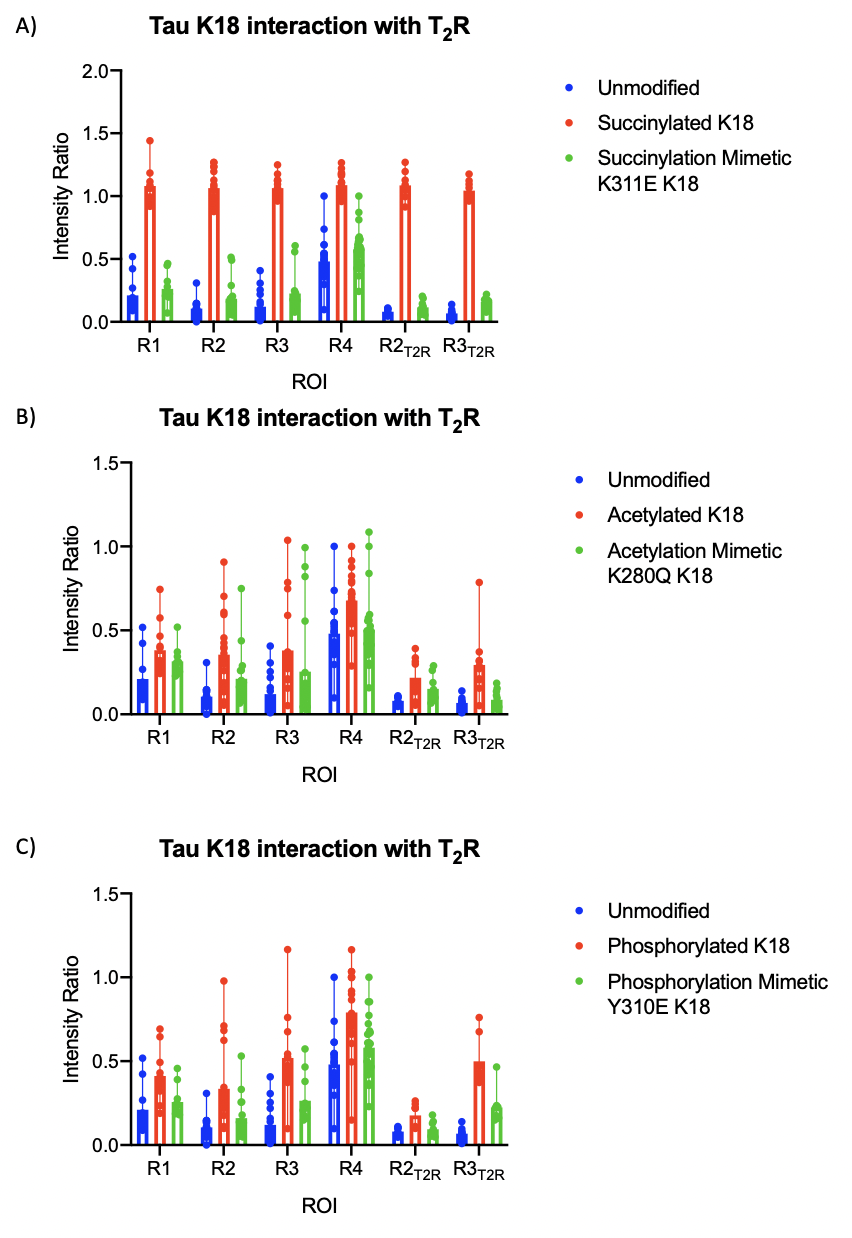


**Figure S6: Comparison of Mimetics to PTM’s on tau-T2R binding.** Intensity ratios were calculated from ^1^H,^15^N HSQC spectra of tau samples (42 uM) with and without T2R (50 uM) and averaged over each MBD repeat as well as the core T2R binding regions R2_T2R_ and R3_T2R._ Intensity ratios of unmodified tau K18 were compared to PTM and mimetics of (A) Succinylation, (B) Acetylation, and (D) Phosphorylation. Data shown as mean (bar) and range (whisker) of all resolved resonances within each repeat region (R1, R2, R3, R4) and core binding regions (R2_T2R_ and R3_T2R_), with individual data points indicated as symbols.


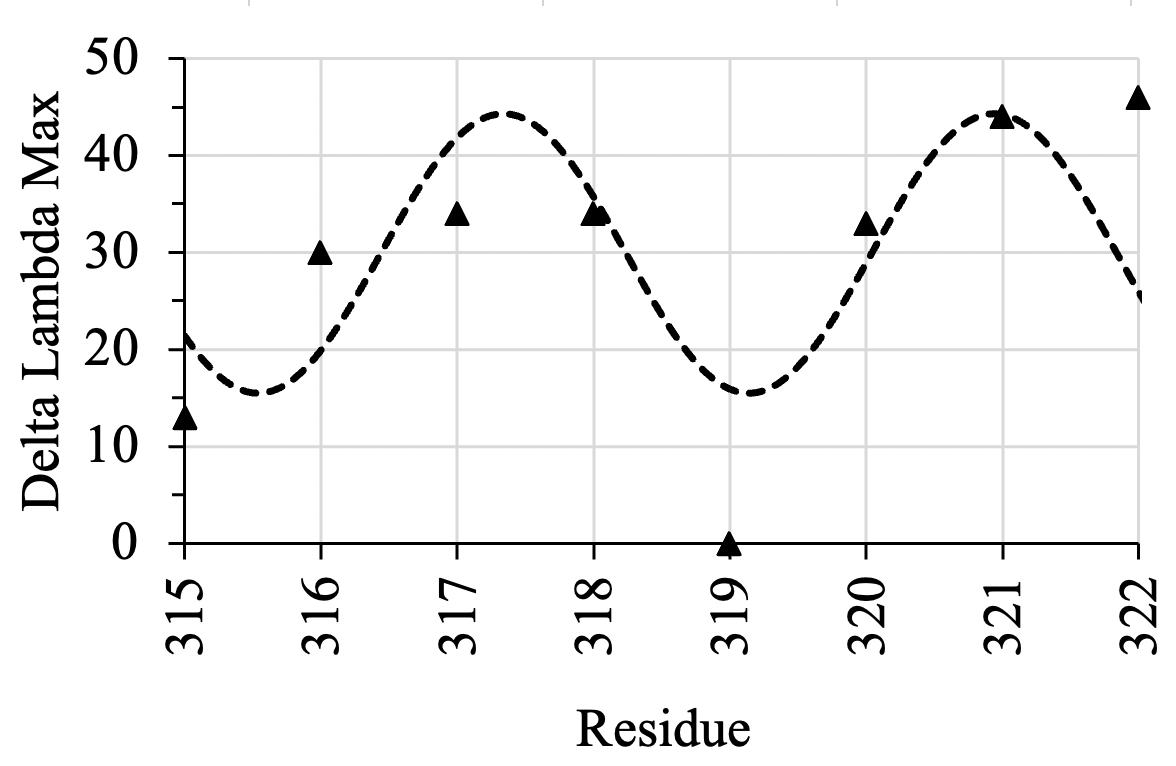


**Figure S7: Acrylodan fluorescence of tau labeled at residues 315-322.** The maximum fluorescence emission wavelength of acrylodan-conjugated single cysteine tau K16 mutants (residues 315-322) was recorded in the presence and absence of lipid vesicles (30 mM PCPS). The difference in the corresponding wavelength for protein in each condition is shown as delta lambda-max. The dashed line represents a fit to the data with a periodicity of 3.6.

Helix 1

Helix 2

Helix 3

Helix 4

A)

B)

C)

D)

**Figure S8: Lipid Binding Curves of Helix 1-4 for K18 and PTM mimetic K18.** Data from ^1^H,^15^N HSQC spectra of tau K18 (62.5 uM) at increasing concentrations (0-25 mM) of 4:1 POPC:POPS lipid vesicles for residues within each helical regions for (A) unmodified K18, (B) acetylation mimetic K280Q, (C) succinylation mimetic K311E, and (D) phosphorylation mimetic Y310E. Data were fit (solid lines) to a one-site model (red) (81).

**Figure S9: Negative stain electron microscopy (EM) of tau oligomers.** Oligomers were isolated from protein/phospholipid complex samples by SEC and visualized by negative stain EM. Complexes were formed with 200 uM of (A) unmodified tau K18, (B) acetylation mimetic K18 K280Q, or (C) succinylation mimetic K18 K311E combined with 4 mM BPS phospholipid vesicles.

**Figure S10: Intensity ratio plots of tau-BPS phospholipid complexes.** Tau protein (200 uM) was incubated with BPS phospholipids (4 mM) overnight. ^1^H,^15^N HSQC spectra were recorded for tau protein monomer and for tau-phospholipid complexes of unmodified tau K18 (A) succinylation mimetic K18 K311E, (B) acetylation mimetic K18 K280Q, and (C) phosphorylation mimetic K18 Y310E (D). Intensity ratios were calculated for assigned resolved resonances, based on previous assignments (58,97), as the intensity of tau-phospholipid complexes normalized by the corresponding intensity in the absence of phospholipids. The regions previously identified as comprising the oligomer core are indicated in grey shading.

**Figure S11: Negative Stain EM images of Tau Fibrils**. 200 uM unmodified tau K18 (TK18) and PTM mimetic tau K18 was incubated with 4 mM BPS for ThT assays. Images were taken at the end of the assay. Scale bars are 100 nm.

**Figure S12: Self-aggregation of tau.** Aggregation of unmodified (A) and mutant (B) K18 at 200 uM concentration was induced with shaking at 1000 RPM at 37°C. Normalized ThT fluorescence was used to monitor fibril formation. T_50_ values, calculated as the time to reach half-maximal fluorescence and T_lag_ values calculated as the time to reach fluorescence units (RFU) >0.1, indicating the start of paired helical filament formation, are tabulated in (C). Data shown are mean and range of three independent experiments, normalized to the final data point at 72 hrs.
